# Momelotinib decreased cancer stem cell associated tumor burden and prolonged disease-free remission period in a mouse model of human ovarian cancer

**DOI:** 10.18632/oncotarget.24615

**Published:** 2018-03-30

**Authors:** Emily Chan, Rodney Luwor, Christopher Burns, George Kannourakis, Jock K. Findlay, Nuzhat Ahmed

**Affiliations:** ^1^ Department of Obstetrics and Gynaecology, University of Melbourne, Victoria 3052, Melbourne, Australia; ^2^ Department of Surgery, Royal Melbourne Hospital, University of Melbourne, Victoria 3052, Melbourne, Australia; ^3^ Walter and Eliza Hall Institute of Medical Research, Victoria 3052, Parkville, Australia; ^4^ Fiona Elsey Cancer Research Institute, Victoria 3353, Ballarat, Australia; ^5^ Federation University Australia, Victoria 3010, Ballarat, Australia; ^6^ The Hudson Institute of Medical Research, Victoria 3168, Clayton, Australia

**Keywords:** ovarian carcinoma, tumor cells, ascites, chemoresistance, chemotherapy

## Abstract

Despite a good initial response to front-line chemotherapy, majority of the ovarian cancer patients relapse with consecutive phases of recurrences; and nearly 60% die within 5 years due to the development of a chemoresistant disease. This study investigated whether inhibition of the Janus kinase 2 (JAK2)-signal transducer and activator of transcription 3 (STAT3) pathway by momelotinib is sufficient in suppressing tumor burden and prolonging the disease-free survival period in a mouse model of ovarian cancer. We demonstrate that paclitaxel treatment enhanced JAK2/STAT3 activation which resulted in the enrichment of cancer stem cell (CSC)-like phenotype in the surviving ovarian cancer cells *in vitro* and in *in vivo* mouse xenografts. Combined treatment with paclitaxel and momelotinib inhibited paclitaxel-induced JAK2/STAT3 activation and CSC-like development in mice xenografts, and consequently reduced the tumor burden significantly greater than that achieved by paclitaxel-treatment alone. However, robust recurrent tumor growth with enhanced JAK2/STAT3 activation and CSC-like phenotype was observed in all mice groups after termination of treatments, but was delayed significantly in the paclitaxel and momelotinib treated group compared to other treatment groups. Daily oral gavage of momelotinib after termination of paclitaxel treatment showed sustained inhibition of tumor growth and a prolonged disease-free survival period in 50% of the mice. The other 50% of mice that developed tumors with ongoing momelotinib treatment also showed significantly increased survival benefit and a smaller tumor burden. These preliminary findings may have a profound clinical impact in developing an effective momelotinib-based ‘maintenance-therapy’ in ovarian cancer patients' post-chemotherapy treatment.

## INTRODUCTION

Despite improvements in the treatment for epithelial ovarian cancer over the last three decades, the mortality rate remains largely unchanged. Majority of the patients who achieve clinical response to paclitaxel and platinum-based chemotherapy experience relapse and die within 5 years of diagnosis [1]. Current chemotherapy treatments lead to substantial tumor regression in the majority of the ovarian cancer cases but they do not eliminate subpopulations of residual cancer stem cells (CSCs) which have been demonstrated to be responsible for the regrowth of tumors in the recurrent scenario [2–6]. Henceforth, recurrence occurs shortly after an otherwise apparently successful first line of treatment. Although targeting CSCs has gained increased popularity as a potential avenue to improve disease-free progression and overall survival in many cancers, the survival mechanisms of CSCs after chemotherapy treatments are still not fully understood [7, 8].

The JAK2/STAT3 pathway mediates the effects of growth factors and cytokines by regulating the expression of downstream gene expression [9]. In normal cells, STAT3 is transiently activated in response to specific growth factors and cytokines (e.g. IL6, GCSF, LIF, EGF). However, in cancers, including breast, ovarian and prostate, STAT3 is constitutively active in certain populations of tumor cells [10, 11]. The STAT3 pathway has been shown to be a prerequisite for the proliferation and maintenance of glioblastoma and breast cancer stem cells [12, 13], rapidly cycling intestinal stem cells [14], as well as ovarian cancer cells [3]. Coupling of the stem cell marker CD44 with the embryonic stem cell marker Nanog has been shown to be associated with the activation of STAT3 in ovarian cancer cells [15]. The activation of STAT3 in these cells resulted in multidrug resistance gene expression and concomitant chemoresistance. We and others have shown significantly enhanced expression of STAT3 in drug-resistant recurrent ovarian tumors derived from metastatic ovarian lesions and ascites-derived tumors, compared to primary tumors and chemonaive ascites-derived tumors [16–18]. The molecular signature of CSCs has also been associated with chemoresistant ovarian and other cancers [18, 19]. Hence, the JAK2/STAT3 and CSC associated pathways are potential targets for the development of novel drugs aimed at suppressing their constitutive as well as ligand induced activation. In the last decade, several anti-STAT3 compounds have been identified to inhibit cancer-associated proliferation, inflammation and chemotherapy resistance [9, 10]. However, none of these compounds have shown effects on CSCs, which theoretically have been suggested to drive chemoresistance.

Previous studies from our group have demonstrated that intraperitoneal (ip) injection of *in vitro* chemotherapy-treated ovarian cancer cells in nude mice resulted in the generation of a larger tumor burden with increased tumor staining of CSC-like cells compared to control untreated cells [3]. Nonetheless, *in vitro* treatment with a combination of chemotherapy and momelotinib (a potent ATP-competitive inhibitor of JAK1/2) substantially suppressed CSC-like cells and tumor burden in mice when these treated-cells were injected in mice [20]. In order to determine the pharmacological and toxicological parameters of chemotherapy and momelotinib treatment *in vivo*, subcutaneous inoculation of untreated ovarian cancer cells was performed in mice, which were subsequently treated ip with paclitaxel with or without daily oral gavage of momelotinib [4]. Consistent daily treatment with momelotinib and weekly paclitaxel resulted in a smaller subcutaneous tumor volume with significant reduction in CSC-like cells in mice xenografts compared to a group of mice treated with weekly paclitaxel alone [4]. The above studies are the first to demonstrate the efficacy of momelotinib as a CSC-targeted therapy for ovarian cancer.

The aim of this study was to evaluate the effect of momelotinib on the CSC phenotype in ovarian cancer cell line models *in vitro* and in an *in vivo* mouse model. The main objective was to evaluate the effect of treatment with momelotinib in combination with paclitaxel on the tumor burden, peritoneal dissemination and disease-free remission period in a mouse model. Two ovarian cancer cell lines representative of high-grade serous (HEY) and clear cell (TOV21G) ovarian carcinomas were chosen to determine the *in vitro* effect of paclitaxel with or without momelotinib. The HEY cell line was further examined in an *in vivo* mouse model to determine the effect of paclitaxel with or without momelotinib. This ‘proof of concept’ study demonstrates that the use of daily oral dosing of momelotinib as a ‘maintenance therapy’ after chemotherapy treatment not only prolongs the disease-free remission period but also inhibits the peritoneal dissemination in a mouse model of ovarian cancer. The findings in this study therefore, warrant future clinical trials for comprehensive evaluation of momelotinib for the better management of ovarian cancer patients.

## RESULTS

### The addition of momelotinib suppressed paclitaxel-induced JAK2/STAT3 pathway activation in ovarian cancer cell lines

**Figure 1:**
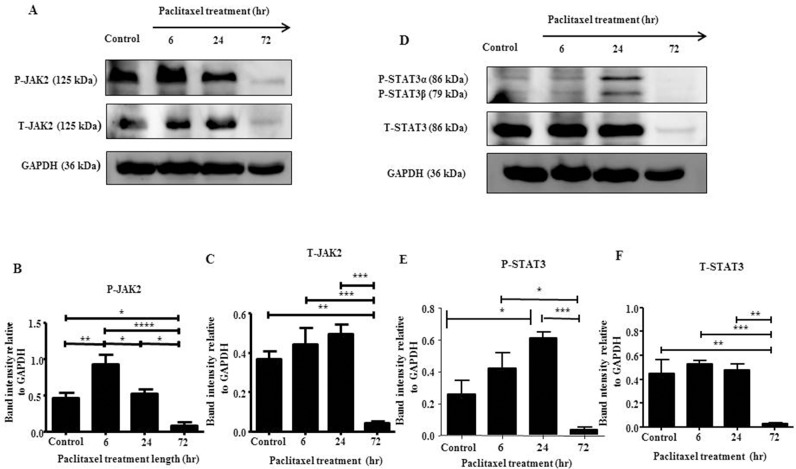
JAK2 and STAT3 activation in HEY cells in response to paclitaxel treatment by Western blot **(A and D)** Total cell lysates of untreated and cells treated with 0.05μg/mL of paclitaxel following 6, 24 and 72 hours of paclitaxel treatment were prepared and subjected to Western blot analysis using antibodies specific for P- or T-JAK2 and P- or T-STAT3. Total protein load was determined by stripping and re-probing the membranes with GAPDH. Images are representative of four independent cell lysate samples. Densitometric analyses of **(B-C)** P-JAK2 and T-JAK2; **(E-F)** P-STAT3 and T-STAT3 protein expression were determined by using Image J. The values represent the relative mean band intensity normalized to GAPDH loading control ± SEM of four independent experiments. Parametric one-way ANOVA with Tukey's post-test was used. Significance is indicated by ^*^p<0.05, ^**^p<0.01, ^***^p<0.001, ^****^p<0.0001.

**Figure 2:**
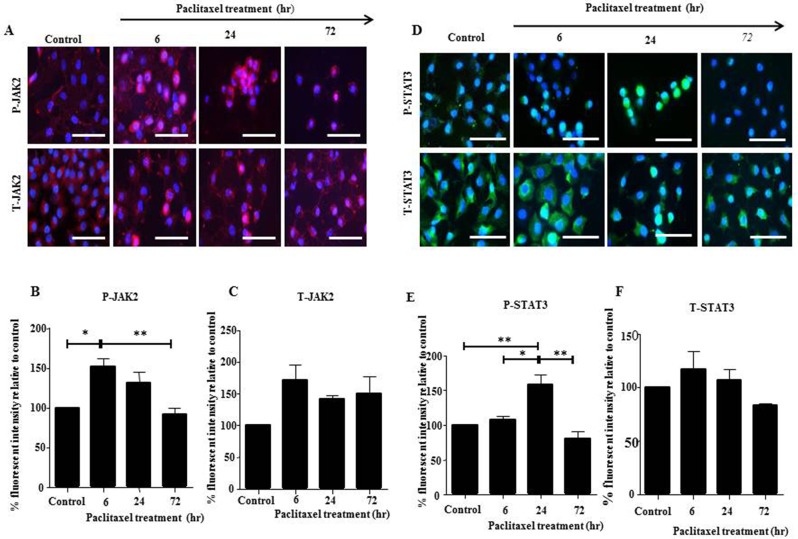
JAK2 and STAT3 activation in HEY cells in response to paclitaxel treatment by immunofluorescence **(A and D)** Expression and localization of activated P-JAK2/STAT3 and T-JAK2 /STAT3 in ovarian cancer HEY cell line following 6, 24 and 72 hours treatment with paclitaxel was determined by immunofluorescence staining. Untreated and treated cells were assessed for the expression of P-JAK2, T-JAK2, P-STAT3 and T-STAT3 by immunofluorescence using rabbit and mouse polyclonal and monoclonal antibodies as described in the Materials and Methods section. Staining was visualized using the secondary Alexa 590 (red) and Alexa 488 (green) fluorescent-labelled antibodies and nuclei were detected by DAPI (blue) staining. Images are representative of three independent experiments. Magnification was 400X; scale bar = 250μm. Quantification of **(B-C)** P-JAK2 and T-JAK2; **(E-F)** P-STAT3 and T-STAT3 fluorescent intensity was determined by using Image J. Results are expressed as the percentage of the average fluorescent intensity value relative to untreated cells ± SEM of three independent experiments. Parametric One-way ANOVA with Tukey's post-test was used. Significance is indicated by ^*^p<0.05, ^**^p<0.01.

In this study, we explored the activation of JAK2/STAT3 pathway in serous HEY and clear cell carcinoma TOV21G cell lines *in vitro* by Western Blot and immunofluorescence in response to the concentration of paclitaxel which inhibited cell growth by 50% (GI50) (HEY: 0.05ng/mL, TOV21G: 0.01ng/mL). HEY cell line demonstrated the highest expression of phosphorylated-JAK2 (P-JAK2) following a 6 hour treatment (Figures 1 and 2, A-C), while phosphorylated-STAT3 (P-STAT3) peaked following a 24 hour treatment (Figures 1 and 2, D-F). For TOV21G cell line, P-JAK2 and P-STAT3 expression began to peak following 24 hours of paclitaxel treatment (Supplementary Figures 1 and 2, A-F). In both HEY and TOV21G cells, P-JAK2 and P-STAT3 proteins were also observed in the nucleus of cells upon activation by paclitaxel (Figure 2, Supplementary Figure 2). These were mostly seen at 6 and 24 hours paclitaxel-treated samples, but were less prominent in 72 hour samples (Figure 2, Supplementary Figure 2). The expression of total (T)-JAK2 and total (T)-STAT3 remained unchanged within 72 hours in response to paclitaxel treatment by immunofluorescence. However, Western blots showed massive down regulation of T-JAK2 and T-STAT3 expression at 72 hours-in HEY cells (Figure 1A and 1D). In TOV21G cells, no change in the expression of total JAK2 and STAT3 was observed by Western blots or immunofluorescence.

**Figure 3:**
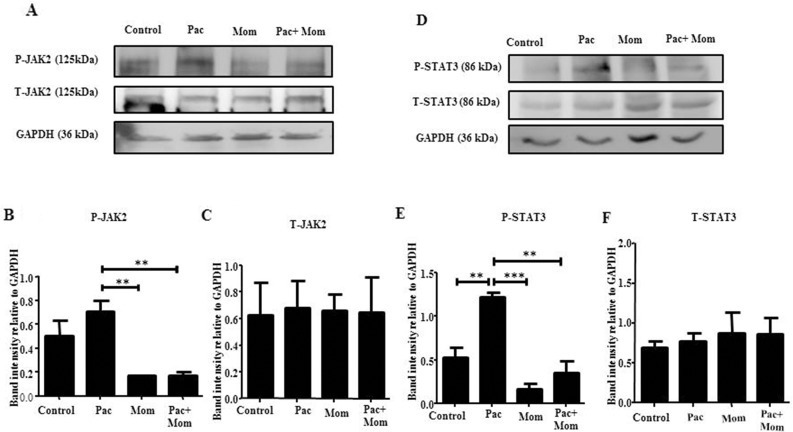
Effect of paclitaxel and/or momelotinib treatment on the activation of JAK2 and STAT3 in HEY cells by Western blot **(A and D)** Total cell lysates of untreated cells and cells treated with 0.05μg/mL of paclitaxel (Pac) with or without 1μM of momelotinib (Mom) for 24 hours were extracted and subjected to immunoblot analysis using antibody specific for P-JAK2/STAT3 or T-JAK2/STAT3. Total protein load was determined by stripping and re-probing the membranes with GAPDH. Images are representative of three independent lysate samples. Densitometric analyses of **(B-C)** P-JAK2 and T-JAK2 and **(E-F)** P-STAT3 and T-STAT3 protein expression were determined by using Image J. The values represent the relative mean of band intensity normalized to GAPDH loading control ± SEM of three independent experiments. Parametric One-way ANOVA with Tukey's post-test was used. Significance is indicated by ^**^p<0.01, ^***^p<0.001.

**Figure 4:**
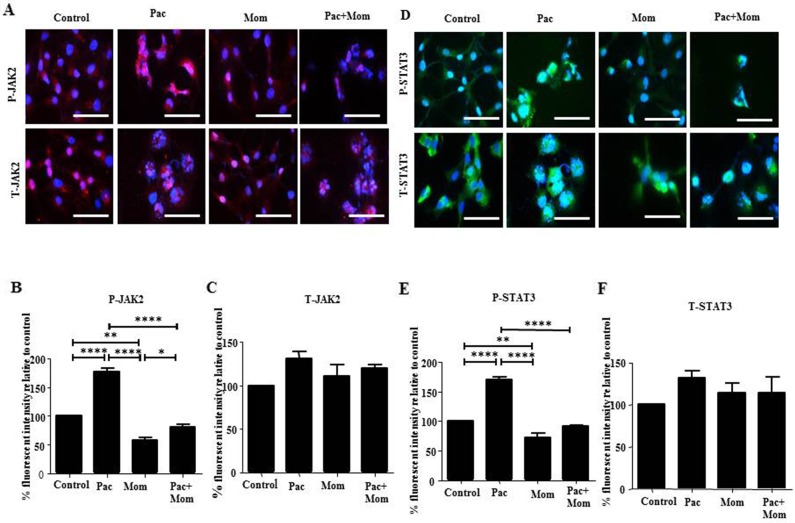
Effect of paclitaxel and/or momelotinib treatment on JAK2 and STAT3 activation in HEY cells by immunofluorescence **(A and D)** Expression and localization of the JAK2 and STAT3 activation in ovarian cancer HEY cells in response to treatments with 0.05μg/mL of paclitaxel (Pac) with or without 1μM of momelotinib (Mom) for 24 hours were determined by immunofluorescence staining. Untreated and treated cells were assessed for the expression of P-JAK2/STAT3 and T-JAK2/STAT3 using rabbit and mouse polyclonal and monoclonal antibodies as described in the Materials and Methods section. Staining was visualized using the secondary Alexa 590 (red) and Alexa 488 (green) fluorescent-labelled antibodies and nuclei were detected by DAPI (blue) staining. Images are representative of three independent experiments. Magnification was 400X; scale bar = 250μm. Quantification of **(B-C)** P-JAK2 and T-JAK2, and **(E-F)** P-STAT3 and T-STAT3 fluorescent intensity was determined using Image J. Results are expressed as the percentage of the average fluorescent intensity value relative to untreated cells ± SEM of three independent experiments. Parametric One-way ANOVA with Tukey's post-test was used. Significance is indicated by ^*^p<0.05, ^**^p<0.01, ^****^p<0.0001.

Optimal inhibition of paclitaxel-induced JAK2/STAT3 phosphorylation was observed with 1 μM momelotinib treatment at 24 hours by Western blot in HEY cells (Supplementary Figure 3A-3D). There was no significant difference in the expression of T-JAK2 and T-STAT3 in all treatment groups (Supplementary Figure 3C-3D). Significant inhibition of JAK2/STAT3 activation by 1μM momelotinib in both HEY and TOV21G cell lines was demonstrated by Western blot and immunofluorescence (Figures 3 & 4) (Supplementary Figures 4 & 5). Momelotinib in the presence of paclitaxel also inhibited the nuclear translocation of P-JAK2 and P-STAT3 to nucleus (Figure 4 and Supplementary Figure 5).

### Momelotinib significantly suppressed the development of CSC-like markers associated with paclitaxel treatment in ovarian cancer cell lines

**Figure 5:**
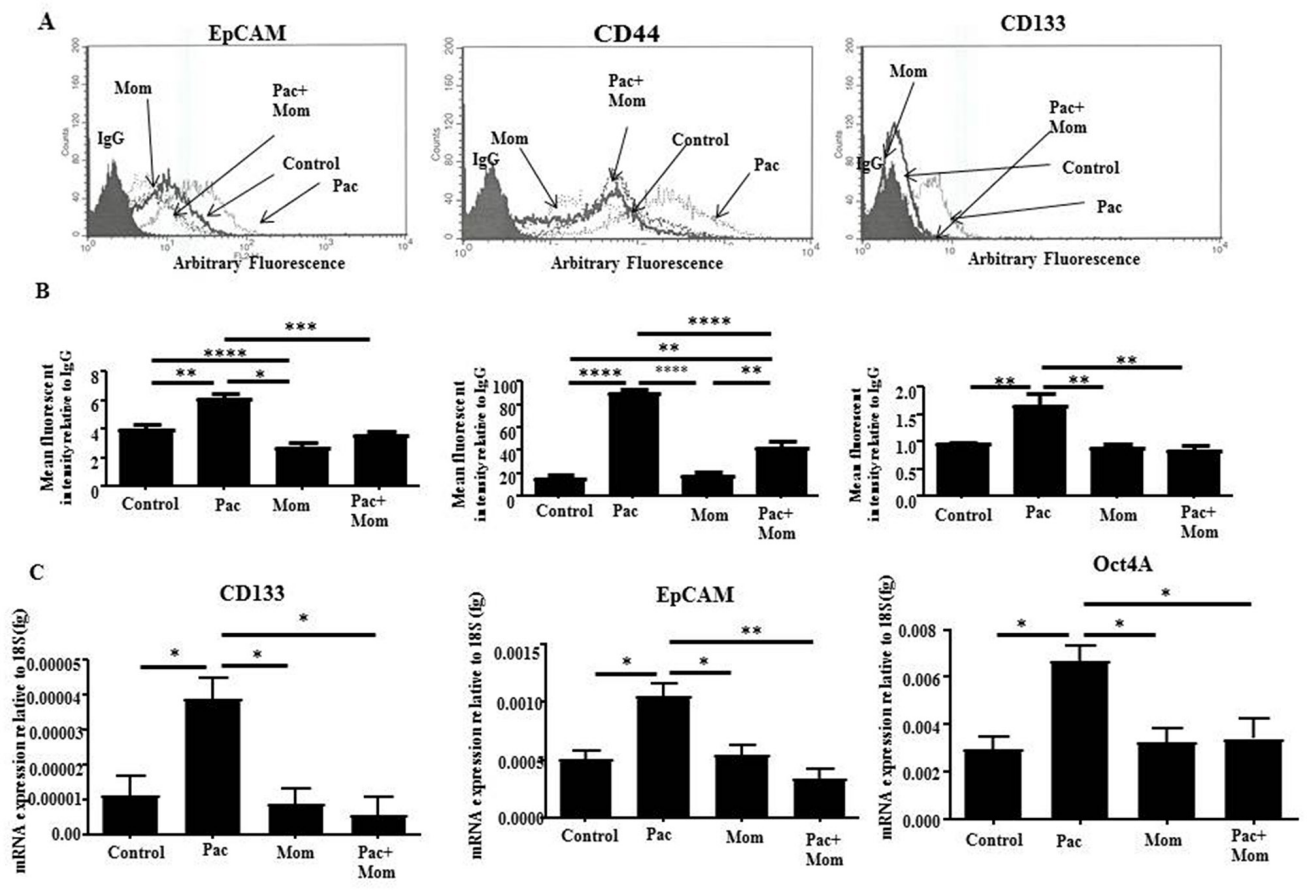
Analysis of the expression of CSC-like surface markers in HEY cells treated with paclitaxel and/or momelotinib **(A)** Cell surface expression of EpCAM, CD44 and CD133 in ovarian cancer HEY cells in response to 24 hours treatments with 0.05μg/ml paclitaxel (Pac), 1μM momelotinib (Mom) or a combination of both (Pac+Mom) were determined by flow cytometry. Untreated and treated cells were incubated with primary antibodies specific for EpCAM, CD44-PE, CD133-PE or IgG, followed by anti-mouse-PE secondary antibody for EpCAM detection. Histograms are representative of four independent experiments. **(B)** Semi-quantitative analysis of the arbitrary fluorescent expression of CSC-like markers was performed. Results are expressed as the mean arbitrary fluorescent expression of the markers of interest relative to negative control IgG ± SEM of four independent experiments. Parametric One-way ANOVA with Tukey's post-test was used. Significance is indicated by ^*^p<0.05, ^**^p<0.01, ^***^p<0.001, ^****^p<0.0001. **(C)** Absolute quantification of the mRNA expression of CSC-like markers in ovarian cancer HEY cells in response to 24 hour treatments with 0.05μg/ml paclitaxel (Pac), 1μM momelotinib (Mom) or a combination of both were determined by qRT-PCR. RNA was extracted from the untreated and treated cells, and qRT-PCR was performed as described in the Materials and Methods section. Results are presented as the mean absolute value of the gene of interest normalized to the 18S housekeeping gene ± SEM of four independent samples in triplicate. Parametric One-way ANOVA with Tukey's post-test was used. Significance is indicated by ^*^p<0.05, ^**^p<0.01.

To evaluate *in vitro* the effects of momelotinib treatment on ovarian CSC-like cells, we studied the changes in the cell surface expression of ovarian CSC markers in HEY and TOV21G cell lines in response to paclitaxel, momelotinib or paclitaxel + momelotinib by flow cytometry. Following paclitaxel treatment, both HEY and TOV21G cell lines demonstrated significant increase in cell surface markers of EpCAM, CD44 and CD133 as observed by flow cytometry, which were significantly inhibited with the addition of momelotinib (Figure 5A and Supplementary Figure 6). This was consistent with the significantly increased mRNA expression of CD133, EpCAM and Oct4A in response to paclitaxel treatment, and their significant reduction with the addition of momelotinib in HEY cells (Figure 5C).

### The effect of momelotinib in a mouse model: A three phase study

#### Phase 1: Daily momelotinib administration in conjunction with weekly paclitaxel significantly reduced *in vivo* expression of CSC markers and associated tumorigenicity of HEY cells in mice

**Figure 6:**
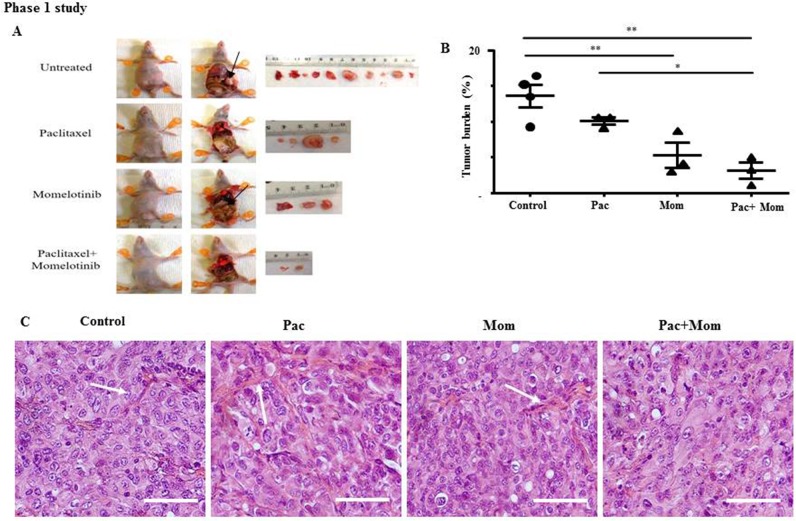
Tumor burden and histology of tumor xenografts derived from mice intraperitoneally injected with HEY cells and treated with either paclitaxel, momelotinib or a combination of both (Phase 1) **(A)** Representative images of mice dissection and resected tumors from control untreated mice and mice treated with weekly paclitaxel, daily momelotinib, or a combination of paclitaxel and momelotinib. All mice treatment was initiated 19 days post-inoculation with HEY cells. At the endpoint of control untreated mice at 35 days post-inoculation, all mice were subjected to euthanasia, dissected and their tumors were excised. Arrows indicate the presence of tumors. **(B)** Quantification of tumor burden is represented by the percentage of the weight of tumor relative to the weight of mice from which the tumor was isolated. Data is presented as mean ± SEM (n=4 control untreated mice, n=3 mice/treated group). Parametric One-way ANOVA with Tukey's post-test was used for statistical analysis. Significance is indicated by ^*^p<0.05, ^**^p<0.01. **(C)** Representative images of H&E stained tumor xenografts derived from control untreated mice and mice treated with either weekly paclitaxel (Pac), daily momelotinib (Mom) or combination of paclitaxel and momelotinib (Pac+Mom) (n=3/group). Arrows indicate the presence of papillary projections. Magnification 400x, scale bar = 10μm.

To investigate the *in vivo* anti-tumorigenic effect following the administration of momelotinib in conjunction with paclitaxel, all mice in Phase 1 study were culled at the endpoint of the control untreated mice and their tumor burden was measured. Thirty-five days post-inoculation of cells, all treated mice were subjected to euthanasia at the humane endpoint of the control untreated mice. Upon dissection, multiple macroscopic tumor deposits of volume (2 - 32 mm^3^) were observed primarily in the liver, pancreas and bowels, and several smaller tumor nodules (0.3 - 1.8 mm^3^) were also found throughout the peritoneal cavity of the control untreated mice (Figure 6A). Mice treated with paclitaxel or momelotinib alone developed macroscopic tumors (2.5 - 22 cm^3^ and 2.5 - 15 mm^3^ respectively) that were found mainly in the liver and bowels, and smaller tumor nodules (0.6 - 1.8 mm^3^ and 0.6 - 1.3 mm^3^ respectively) seen scattered throughout the peritoneal cavity. Mice treated with a combination of paclitaxel and momelotinib developed small tumor nodules (0.01 - 1.3 mm^3^) and fewer macroscopic tumors (2 - 6.4 mm^3^) that were discovered primarily in the bowels. Overall, fewer tumors were found and isolated from each mice in the treatment group compared to control untreated mice (Figure 6A).

We next compared the tumor burden between all mice in Phase 1. Control untreated mice developed the largest tumor burden compared to all groups, with a mean of 13.6 ± 1.6% standardised to body weight (Figure 6B). No significant difference was observed in the average tumor burden between mice that were treated with weekly paclitaxel compared to control untreated mice. However, mice treated with the combination had significantly smaller tumor burden (3.2 ± 1.1%) compared to mice treated with weekly paclitaxel (10.1 ± 0.5%) and the control group (13.6+1.6%). Paclitaxel, momelotinib or a combination of both treatments were well tolerated in mice without any adverse toxicological signs throughout the experiment. No significant difference in the average body weight was observed between all groups throughout this study.

Histological examination of the tumor xenografts revealed morphological features resembling high grade serous carcinoma in women with ovarian cancer (Figure 6C). In general, the isolated mouse tumors from all groups displayed extensive cellular budding, aneuploidy, very large and rounded nuclei and branches of stromal and papillae structures. Diffuse papillary projections with large irregular size and disseminated pattern suggestive of malignancy were seen commonly in the tumors isolated from paclitaxel-treated mice compared to all other groups, while a much lesser extent of papillary projection was seen in the tumors of mice treated with a combination of paclitaxel and momelotinib.

**Figure 7:**
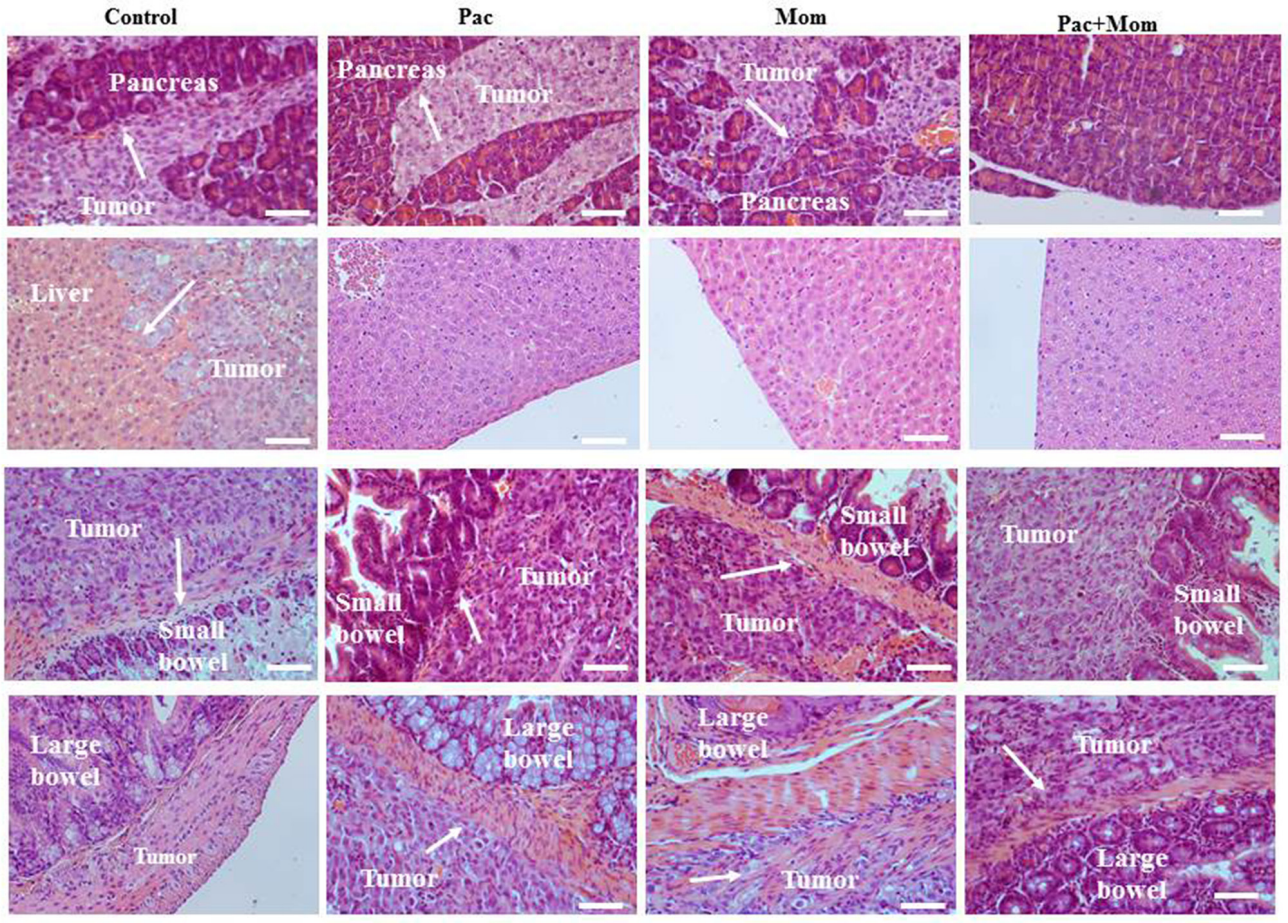
Histological assessment of pancreatic, hepatic, small and large bowel infiltration by tumors developed in mice inoculated with HEY cells in Phase 1 Representative images of H&E stained pancreas, liver, small and large bowel derived from untreated mice and mice treated with either weekly paclitaxel (Pac), daily momelotinib (Mom) or combination of paclitaxel and momelotinib (Pac+Mom) (n=3/group). Arrows indicate tumor cells invading respective organs. Magnification 200x, scale bar = 10μm.

To determine whether the administration of paclitaxel and momelotinib had any *in vivo* effect on tumor metastasis, mice organs (liver, pancreas, spleen, kidney, small bowel and large bowel) were collected and subjected to H&E staining. Tumor invasion was seen in the pancreas and bowels of control mice and those treated with either weekly paclitaxel or daily momelotinib. However, liver invasion was seen only in the control mice group. Mice that were treated with a combination of weekly paclitaxel and daily momelotinib had tumor invasion that involved only the small bowel (Figure 7). Although tumors were visible on the kidney wall of untreated and paclitaxel-treated mice, no visible tumor infiltration was seen.

**Figure 8:**
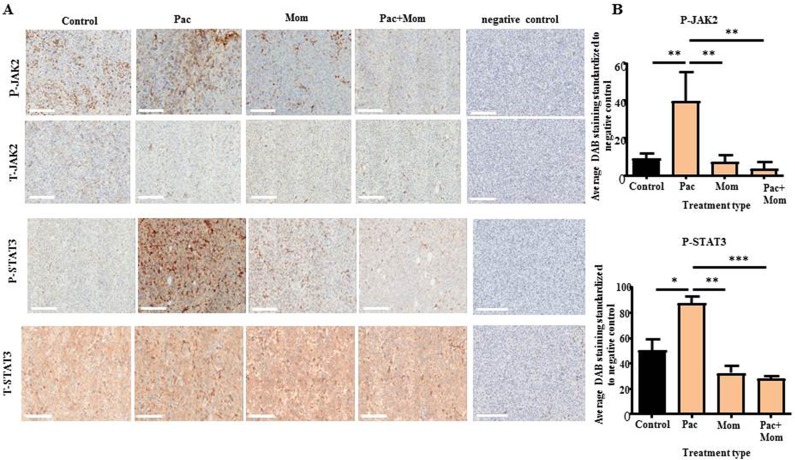
Immunohistochemical analysis of P-JAK2, T-JAK2, P-STAT3 and T-STAT3 expression in tumor xenografts derived from mice intraperitoneally injected with HEY cells in Phase 1 **(A)** Representative images of P-JAK2, T-JAK2, P-STAT3 and T-STAT3 immunohistochemistry staining in paraffin embedded tumor xenografts derived from control untreated mice, and mice treated with either weekly paclitaxel (Pac), daily momelotinib (Mom) or a combination of paclitaxel and momelotinib (Pac+Mom). Magnification 200x, scale bar = 200μm. **(B)** Quantification of P-JAK2 and P-STAT3 DAB staining was performed using Fiji software as described in the Materials and Methods. Results are expressed as the average DAB reading of positively-stained tumor cells subtracted by the average DAB staining of the negatively-stained cells for each xenograft ± SEM (n=3/group). Parametric One-way ANOVA with Tukey's post-test was used. Significance is indicated by ^*^p<0.05, ^**^p<0.01, ^***^p<0.001.

Immunohistochemistry analysis of the xenografts of mice treated with paclitaxel showed significantly higher P-JAK2 and P-STAT3 expression when compared to the untreated group, while no significant difference was observed for T-JAK2 and T-STAT3 staining between all groups (Figure 8). Treatment with paclitaxel + momelotinib or momelotinib on its own resulted in tumors that expressed significantly reduced P-JAK2 and P-STAT3 when compared to the group that received paclitaxel treatment.

**Figure 9:**
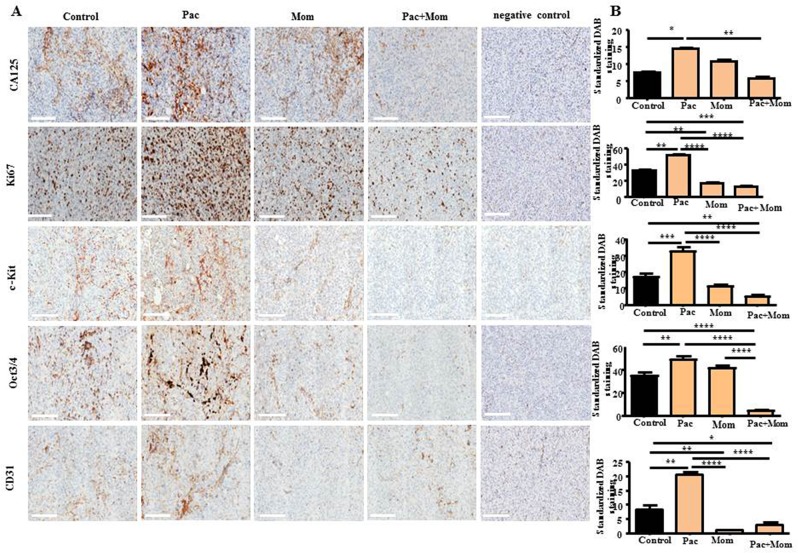
Immunohistochemical analysis of CA125, Ki67, c-Kit, Oct3/4 and CD31 expression in tumor xenografts derived from mice intraperitoneally injected with HEY cells in Phase 1 **(A)** Representative images of CA125, Ki67, c-Kit, Oct3/4 and CD31 immunohistochemistry staining in paraffin embedded tumor xenografts derived from control untreated mice and mice treated with either weekly paclitaxel (Pac), daily momelotinib (Mom) or a combination of paclitaxel and momelotinib (Pac+Mom). Magnification 200x, scale bar = 200μm. **(B)** Quantification of CA125, Ki67, c-Kit, Oct3/4 and CD31 DAB staining was performed using Fiji software as described in the Materials and Methods. Results are expressed as the average DAB reading of positively-stained tumor cells subtracted by the average DAB staining of the negatively-stained cells for each xenograft ± SEM (n=3/group). Parametric One-way ANOVA with Tukey's post-test was used. Significance is indicated by ^*^p<0.05, ^**^p<0.01, ^***^p<0.001, ^****^p<0.0001.

To further analyse the tumor phenotype following administration of momelotinib and paclitaxel treatment in mice, the resected tumors of mice in Phase 1 were subjected to immunohistochemical analysis for the expression of cancer antigen 125 (CA125), proliferative marker Ki67, cluster of differentiation 31 (CD31), and CSC-like markers c-Kit and Oct3/4 (Figure 9). Significant increase in CA125, Ki67 and CD31 staining was observed in the xenograft derived from paclitaxel-treated mice compared to the xenograft of control untreated mice (Figure 9). Consistent with these results, tumors from the paclitaxel-treatment group had significantly increased expression of c-Kit and Oct3/4 compared to the untreated control group (Figure 9). On the other hand, the addition of momelotinib to paclitaxel significantly reduced CA125 tumor staining by 2.5-fold when compared to the xenografts of mice treated with paclitaxel alone. Mice that received daily momelotinib alone or in conjunction with weekly paclitaxel had tumors that exhibited significantly reduced Ki67 and CD31 staining when compared to that of the control untreated mice and paclitaxel-treated mice (Figure 9). Tumors derived from the group that received momelotinib treatment showed a reduction in c-Kit expression compared to paclitaxel-treated groups. The group that received paclitaxel + momelotinib produced tumors that displayed a significant reduction in c-Kit and Oct3/4 staining when compared to that of control untreated and paclitaxel-treated mice (Figure 9).

#### Phase 2: Termination of paclitaxel, momelotinib or a combination of both treatments resulted in robust tumor development

**Figure 10:**
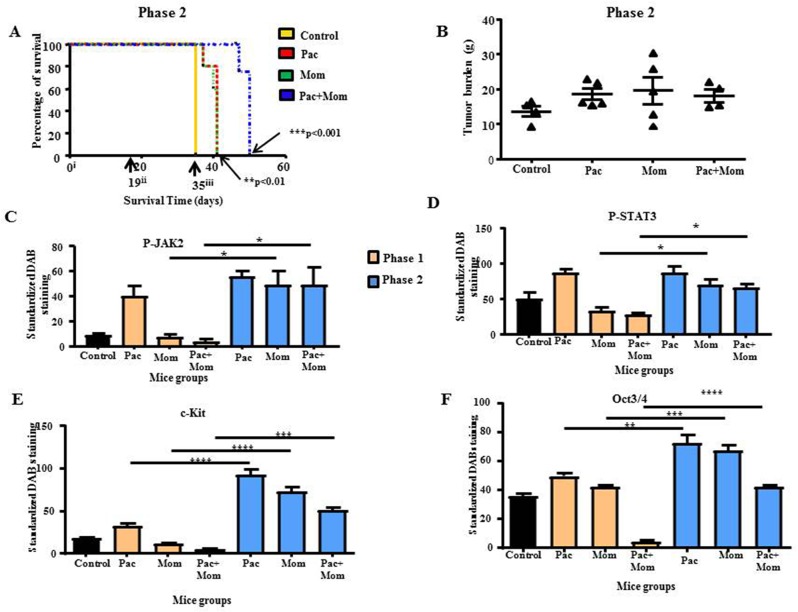
Survival analysis and tumor burden of mice in Phase 2 study; quantitative comparison of P-JAK2, P-STAT3, c-Kit and Oct3/4 immunohistochemical analysis of tumor xenografts derived from mice in Phase 1 and 2 studies **(A)** Kaplan-Meier survival analysis of control untreated mice (yellow line), mice treated with either weekly paclitaxel (red dotted line), daily momelotinib (green dotted line) or a combination of paclitaxel and momelotinib (blue dotted line) in Phase 2. Parametric One-way ANOVA with Tukey's post-test was used. Significance is indicated by ^**^p<0.01, ^***^p<0.001. **(B)** Quantification of tumor burden is represented by the percentage of the weight of tumor (g) relative to the weight of mice (g) from which the tumor was isolated. Data is presented as mean ± SEM (n=4 control untreated mice, n=5 treated mice/group). **(C-F)** Comparative quantification of P-JAK2, P-STAT3, c-Kit and Oct3/4 DAB staining in mice tumor xenografts between Phase 1 and 2. Results are expressed as the average DAB reading of positively-stained tumor cells subtracted by the average DAB staining of the negatively-stained cells for each xenograft ± SEM (n=3/group). Parametric One-way ANOVA with Tukey's post-test was used to compare between the similar mice groups of Phases 1 and 2. Significance is indicated by ^*^p<0.05, ^**^p<0.01, ^***^p<0.001, ^****^p<0.0001.

In Phase 2, mice were treated as in Phase 1, and termination of all treatments was performed at the endpoint of Phase 1. These mice were then allowed to survive until the experimental endpoint of each mouse was achieved. The survival period, tumor burden and dissemination of tumor in mice were noted in Phase 2. Overall, all treated-groups survived longer than the control untreated group (Figure 10A). Mice treated with a combination of paclitaxel and momelotinib survived the longest when compared to all groups, 2 weeks longer compared to control untreated mice. While the difference between paclitaxel only treated and momelotinib only treated mice was not significant, both these groups survived five days longer compared to the control untreated group (Figures 10A).

Termination of paclitaxel, momelotinib or a combination of both treatments resulted in the development of multiple tumors of varying sizes throughout the peritoneal cavity similar to that observed in control untreated mice. Overall, there was no significant difference in the average tumor burden between all groups in Phase 2 (Figure 10B). Similar to Phase 1 study, the morphological features of tumors in mice resembled high grade serous carcinoma in women (Supplementary Figure 7A). The presence of larger stromal projections was more common in the tumors isolated from mice with terminated paclitaxel and terminated momelotinib treatment, while fewer and smaller stromal structures were seen in the tumors of mice with terminated combined treatments of paclitaxel and momelotinib. In addition to the bowel invasion observed in Phase 1, tumor invasion into the liver and pancreas was also observed in mice with terminated paclitaxel or momelotinib alone. On the other hand, tumor invasion was observed in the pancreas, spleen and bowels of mice in which paclitaxel+momelotinib treatment was terminated (Supplementary Figure 7B).

Overall, immunohistochemical staining of xenografts revealed significant increase in P-JAK2 and P-STAT3 staining in the xenografts of mice with terminated momelotinib or a combination of paclitaxel and momelotinib when compared to their respective groups in the Phase 1 study (Figures 10C, 10D and Supplementary Figure 8). However, no statistical significance was detected between the paclitaxel groups in both Phase 1 and Phase 2 studies and between the three treatments terminated groups in Phase 2 (Figures 10C and 10D). No significant difference was detected in the tumor staining of T-JAK2 and T-STAT3 between all groups in Phase 1 and 2 studies.

Consistent with P-JAK2/P-STAT3 staining, immunohistochemical analysis for CSC markers c-Kit and Oct3/4 as well as Ki67 and CD31 were found to be significantly enhanced in the xenografts derived from mice with terminated paclitaxel, momelotinib, and paclitaxel+momelotinib compared to their respective groups in Phase 1 study (Figures 10E, 10F and Supplementary Figures 8 and 9). The tumor staining of CA125 was significantly higher in mice with terminated paclitaxel compared to the paclitaxel group in Phase 1; but remained unchanged between the paclitaxel+momelotinib groups in Phase 2 and Phase 1 studies (Supplementary Figure 9). No significant difference in c-Kit, Oct3/4, Ki67, CD31 and CA125 staining was detected between the three treatment-terminated groups in Phase 2 study (Figures 10E, 10F and Supplementary Figures 8 and 9).

#### Phase 3: Continuous administration of momelotinib after termination of paclitaxel treatment significantly enhanced the disease-free survival time in 50% of mice

**Figure 11:**
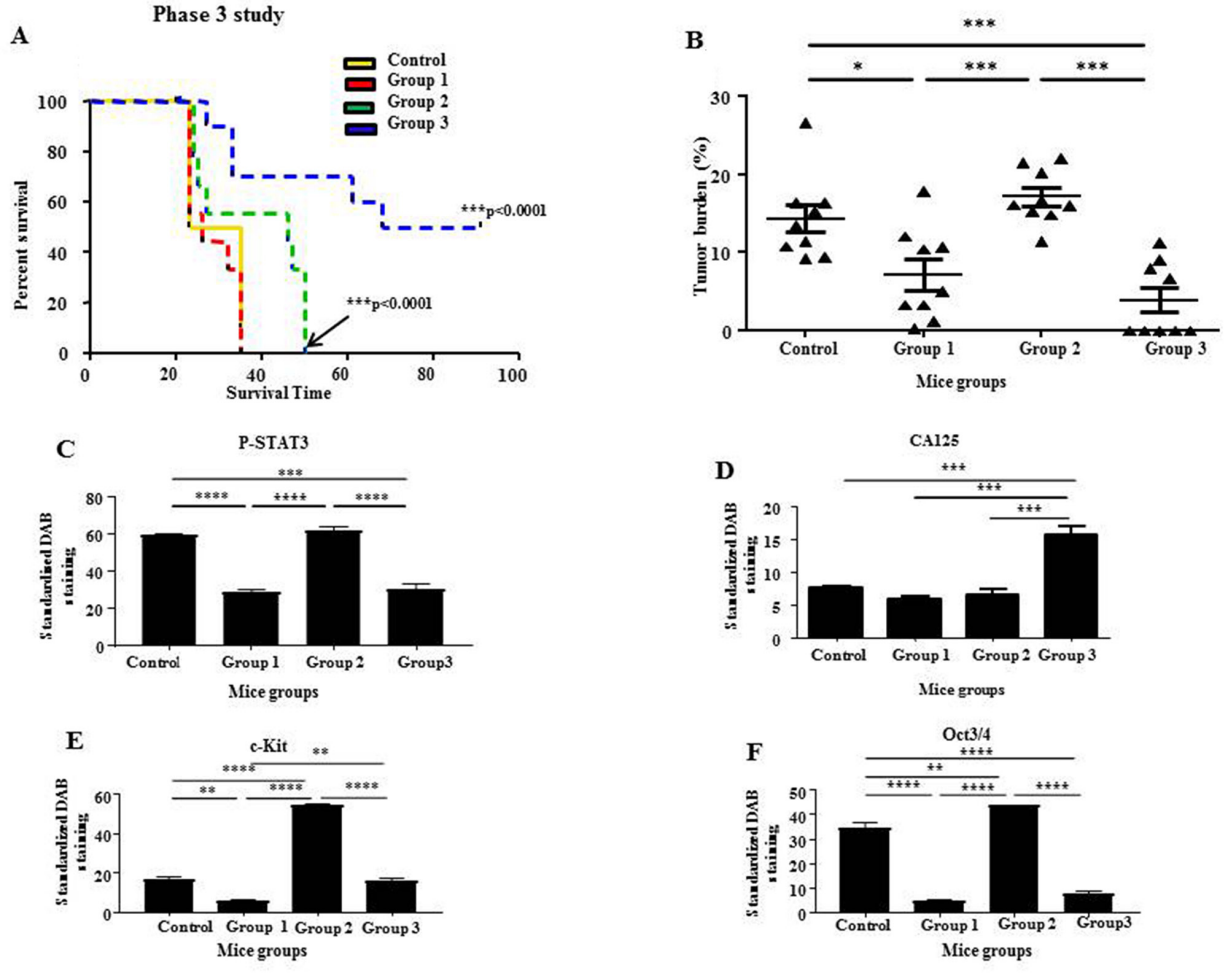
Survival analysis and tumor burden of mice with ongoing momelotinib treatment in Phase 3; quantitative comparison of P-STAT3, CA125, c-Kit and Oct3/4 immunohistochemical analysis of tumor xenografts derived from mice in Phase 3 **(A)** Kaplan-Meier survival analysis of control untreated mice (yellow line), Group 1 mice (red dotted line), Group 2 mice (green dotted line) and Group 3 mice (blue dotted line). All mice were treated with a combination of paclitaxel and momelotinib. Group 1 mice were culled at the endpoint of control untreated mice. Groups 2 and 3 mice were followed until they reached their individual endpoints or until the designated endpoint of this study at 91 days post-inoculation. Parametric One-way ANOVA with Tukey's post-test was used. Significance is indicated by ^***^p<0.001. **(B)** Quantification of tumor burden is represented by the percentage of the weight of tumor (g) relative to the weight of mice (g) from which the tumor was isolated. Data is presented as mean ± SEM (n=9/group). Parametric One-way ANOVA with Tukey's post-test was used. Significance is indicated by ^*^p<0.05, ^***^p<0.001. **(C-F)** Quantification of P-STAT3, CA125, c-Kit and Oct3/4 DAB staining in paraffin embedded tumor xenografts derived from mice in Phase 3. Results are expressed as the average DAB reading of positively-stained tumor cells subtracted by the average DAB staining of the negatively-stained cells for each xenograft ± SEM (n=3/group). Parametric One-way ANOVA with Tukey's post-test was used to compare between the similar mice groups of Phase 3. Significance is indicated by ^**^p<0.01, ^***^p<0.001, ^****^p<0.0001.

In Phase 3, Groups 2 and 3 mice survived significantly longer than control and Group 1 mice (Figure 11A). Kaplan Meier analysis of survival revealed that control untreated mice survived 23-35 days post-inoculation of HEY cells, Group 1 mice survived 25-35 days, while Group 2 mice with terminated paclitaxel and momelotinib-combined treatment survived 24-50 days post-inoculation of HEY cells (Figure 11A). Interestingly, Group 3 mice with terminated paclitaxel but ongoing daily momelotinib treatment survived 33-91 days post-inoculation, with 50% of mice surviving until the designated endpoint at 91 days post-inoculation. Four out of the nine mice in Group 3 that received ongoing momelotinib treatment developed tumors and subsequently survived 33-68 days post-inoculation with HEY cells (Figure 11A). However, the other 5 out of 9 mice survived until the designated endpoint at 91 days post-inoculation. Only one small tumor nodule was found on the bowel of one mouse, while no tumor was seen in the rest of the mice in this group that survived 91 days post HEY cell inoculation. Overall, Group 3 mice that received ongoing daily momelotinib developed an average tumor burden that was significantly smaller compared to the average tumor burden generated in control untreated mice and Group 2 mice (>3 fold less in both cases) (Figure 11B). The tumor burden in Group 1 was also significantly smaller than the tumor burden in the control group (approximately 2-fold). However, no significant difference in the tumor burden was observed between the control and Group 2 mice and Groups 1 and 3 mice (Figure 11B).

Immunohistochemical analysis of P-STAT3, T-STAT3, CA125, c-Kit and Oct3/4 performed on the resected tumors of Group 3 mice was compared to the tumor staining of control untreated mice and mice from Groups 1 and 2 (Figures 11C-11F). The tumor staining of P-STAT3 in Group 3 mice was significantly lower when compared to the tumor staining in control untreated mice and Group 2 mice, while no significant difference was observed when compared to Group 1 mice (Figure 11C). No significant difference was detected in T-STAT3 staining between all groups (Supplementary Figure 10). Ongoing momelotinib treatment in Group 3 mice resulted in tumors with significantly reduced c-Kit and Oct3/4 staining when compared to Group 2 mice (Figures 11E and 11F). However, the average DAB staining of CA125 in Group 3 mice was significantly enhanced when compared to control untreated and mice in Groups 1 and 2 (Figure 11D).

## DISCUSSION

Targeted agents have been the primary focus in enhancing the treatment regime for patients with recurrent ovarian cancer [21]. We have previously shown that ascites-derived ovarian tumor cells isolated from recurrent patients treated with chemotherapy have significantly higher expression of activated JAK2/STAT3 pathway compared to the tumor cells isolated from the ascites of chemonaive patients [22]. In this study, we show a similar up-regulation of activated JAK2/STAT3 pathway, and concomitant significant increase in the expression of CSC-like markers in HEY and TOV21G ovarian cancer cell lines in response to paclitaxel treatment *in vitro*. Paclitaxel-induced JAK2/STAT3 activation was inhibited significantly by momelotinib, which concurrently inhibited the CSC-like marker expression in both HEY and TOV21G cell lines. These results suggest an association between the activation of JAK2/STAT3 pathway and the emergence of CSCs in ovarian cancer cells in response to paclitaxel treatment. In addition to the *in vitro* studies, we also demonstrate that paclitaxel treatment significantly enhanced the activation of the JAK2/STAT3 pathway and CSC-like phenotype in the xenografts of mice injected with a serous human ovarian cancer HEY cell line. Despite a reduction in the tumor burden, paclitaxel treatment generated an aggressive tumor phenotype in HEY cell injected mice, as evidenced by significantly enhanced CA125, Ki-67 and CD31 expression in mice xenografts when compared to that of control untreated mice. As a result, the disease-free survival period of mice after termination of paclitaxel treatment was short-lived, due to the increase in tumor burden in mice. These scenarios are frequently mimicked in the clinical settings where high-grade serous ovarian cancer patients develop highly aggressive recurrent disease within a short time span after successful front line chemotherapy treatment [23].

The addition of daily momelotinib to weekly paclitaxel treatment significantly inhibited the JAK2/STAT3 activation and CSC-like phenotypes in mice tumors. This was consistent with the significantly reduced tumor burden and intraperitoneal tumor metastasis in mice. However, the discontinuation of paclitaxel and momelotinib treatment no longer suppressed JAK2/STAT3 activation and CSC-like development, thus allowing the resumption of tumor growth and dissemination. On the other hand, ongoing suppression of JAK2/STAT3 activation with daily momelotinib administration in Phase 3 as a ‘maintenance therapy’ substantially reduced the tumor burden, and drastically improved the disease-free survival in 50% of the mice; while the other 50% mice demonstrated significantly less tumor burden compared to control and Group 2 mice tumors and had a significantly prolonged disease-free survival period. These observations support the notion that a chemotherapy-triggered microenvironment provokes the escalation of resistant progenies that express the activated JAK2/STAT3 pathway with CSC-like arsenals to tackle the cytotoxic effects of chemotherapy; while cells expressing subtle/low JAK2/STAT3 activation succumb to chemotherapy treatment. However, if the suppression of an activated JAK2/STAT3 pathway can be sustained by momelotinib, tumor growth can be repressed which can result in a prolonged disease-free survival period. In this context, a recent manuscript has demonstrated large primary tumors and wide-spread metastases in mice injected with ascites-derived ovarian cancer cells with high P-STAT3 status compared to mice injected with low P-STAT3 status of ascites derived tumor cells [24]. These studies further support the association between elevated P-STAT3 signalling and the facilitation of ovarian tumorigenesis. Hence, combination therapies that include JAK2/STAT3 and CSC-specific targeted agents and a non-CSC-targeted agent such as chemotherapy, may be a more effective approach for the treatment of ovarian cancer [25–27].

The first identification of ovarian CSC was reported in the ascites of an ovarian cancer patient, which was derived from a single cell and was shown to sequentially propagate tumors over several generations [28]. Since then CSCs have been isolated from ovarian tumors and cell lines by using identifiable cell surface and non-surface markers commonly used in solid malignancies. CSCs in these studies have been isolated depending on the distinct pattern of surface markers such as CD44, EpCAM, CD133, CD117 (c-Kit), Thy1, CD24, etc. [2, 7, 29–33] and non-surface markers, such as aldehyde dehydrogenase [34]. Even though CSCs sorted on the basis of these markers have shown the potential to have ‘CSC characteristics’ (such as, ability to self-renew, resistance to therapy, develop tumors with as low as 100 cells, etc), their significance in the clinical setting remains unknown. Among the CSC markers, CD44, EpCAM, c-Kit, CD133, Oct3/4 have been shown to be abundantly expressed in ovarian carcinomas, metastatic and recurrent tumors. Additionally, their high expression has been correlated with resistance to chemotherapy, shorter disease-free and overall survival [2, 7, 29, 35]. Clinical trials using monoclonal anti-human antibodies and small molecule inhibitors against some of these CSC-associated markers are in progress but their application in the clinical setting remains unknown [2, 7, 29]. In this study, CD44, EpCAM, c-Kit and CD133 were used as CSC markers to study the responses of paclitaxel and/or momelotinib treatments in *in vitro* ovarian cancer cell lines. However, most of the antibodies used for the *in vitro* characterization of these CSCs could not be used for mouse xenografts due to their lack of specificity on paraffin-embedded tumor sections, resulting in either a strong background staining or no signal on most xenografts. Hence, an effort was made to analyze and quantify the markers (such as CA125, CD31, Ki67, c-Kit and Oct3/4) which would give meaningful results consistent with ovarian cancer progression.

CA125 (also known as MUC16) is a type-1 transmembrane glycoprotein that is up-regulated in the blood and tumors of many cancer patients, including ovarian cancer and is associated with poor prognosis [36, 37]. CA125 is routinely used to diagnose, screen and predict treatment outcomes in ovarian cancer patients [36, 38]. CA125 know down studies in breast, ovarian and pancreatic cancers have shown its association in imparting tumorigenic, metastatic, chemoresistant and anti-apoptotic functions in cancer cells [39–44]. In addition, the ectopic expression of different lengths of carboxyl terminal fragments of CA125 in cancer cells has shown to increase metastatic and chemoresistant properties, suggesting that these fragments may be crucial in generating these functions in cancer cells [42, 45]. A recent study has demonstrated endogenous expression of a carboxy terminal cleaved 17 kDa MUC16-Cter fragment in normal human bronchial cells [45]. Forced expression of a 17 kDa MUC16-Cter fragment in pancreatic cancer cells mediated nuclear translocation of JAK2 which preferentially phosphorylated tyrosine 41 of histone H3 (H3Y41) in a STAT-independent manner and up regulated stemness specific genes *LMO2* [45, 46] and *NANOG* [46, 47]. MUC16-Cter also mediated ALDH^+^ cancer stem cell enrichment which induced tumorigenic, metastatic and drug resistant properties in pancreatic cancer cells [48]. Even though, a non-canonical translocation of activated JAK2 to nucleus was observed in response to paclitaxel treatment in HEY cells, it remains unknown if paclitaxel-induced JAK2 translocation to nucleus is associated with the increased expression of a carboxy-terminal fragment of CA125 associated with CSC specific genes.

The reduction of tumor growth in a mouse model with the addition of a novel inhibitor for JAK2 kinase, momelotinib, had a significant effect on the expression of CD31 in resulting xenografts. We demonstrate that paclitaxel-primed surviving tumor cells not only progress towards CSC-like cells but also drive tumor vascularisation as portrayed by enhanced CD31 expression and dominant papillary projections in paclitaxel-treated mice xenografts. However, the addition of momelotinib to paclitaxel impaired that effect as indicated by the reduction in CD31 expression and less stromal diffusion by histology. This may be one of the driving factors that significantly delays tumor progression in the paclitaxel+momelotinib group, compared to the paclitaxel or momelotinib treatment groups in Phase 2 study and in the ongoing momelotinib treatment group in Phase 3 study. Even though these results are based on a mouse model using one ovarian cancer cell line, it augments valuable information about the effect of momelotinib in combination with paclitaxel on intraperitoneal ovarian tumor progression, and provides a stepping stone for future studies using other ovarian cancer cell lines and ideally primary ovarian cancer cells isolated from ovarian tumors and ascites of ovarian cancer patients.

## CONCLUSION

This study is the first to describe the effectiveness of momelotinib, where prolonged daily treatment of momelotinib by oral gavage as a ‘maintenance therapy’ significantly improved the disease-free and overall survival in a mouse model of human ovarian cancer. The fact that 50% of mice were tumor-free for 91 days post HEY cell transplantation by daily dosing of momelotinib after paclitaxel treatment had terminated, indicates a remarkable improvement in the disease-free and overall survival. In addition, the other 50% of mice that developed tumors while still receiving daily momelotinib treatment had significantly reduced tumor burden compared to control and Group 2 mice. These mice also had a significantly greater overall survival compared to mice in Phase 2 where the treatment was terminated. This demonstrates that the ongoing momelotinib treatment effectively maintained the suppression of tumor growth and progression in mice. Nonetheless, the 50% unresponsive mice group, presumably resistant to momelotinib treatment, that developed tumors showed levels of JAK2/STAT3 activation, c-Kit and Oct3/4 expression that were similar to their paclitaxel+ momelotinib counterparts in Phase 1 which also showed similar tumor burden. Resistance to targeted therapies in a sub-population of cancer patients remains a major problem despite initial clinical responses [49, 50]. The mechanisms underlying resistance to momelotinib therapy may be due to activation of an alternative complementary pathway which may support the survival of momelotinib-resistant population. Cross-talks between signalling pathways have been demonstrated in many tumors [51].

**Figure 12:**
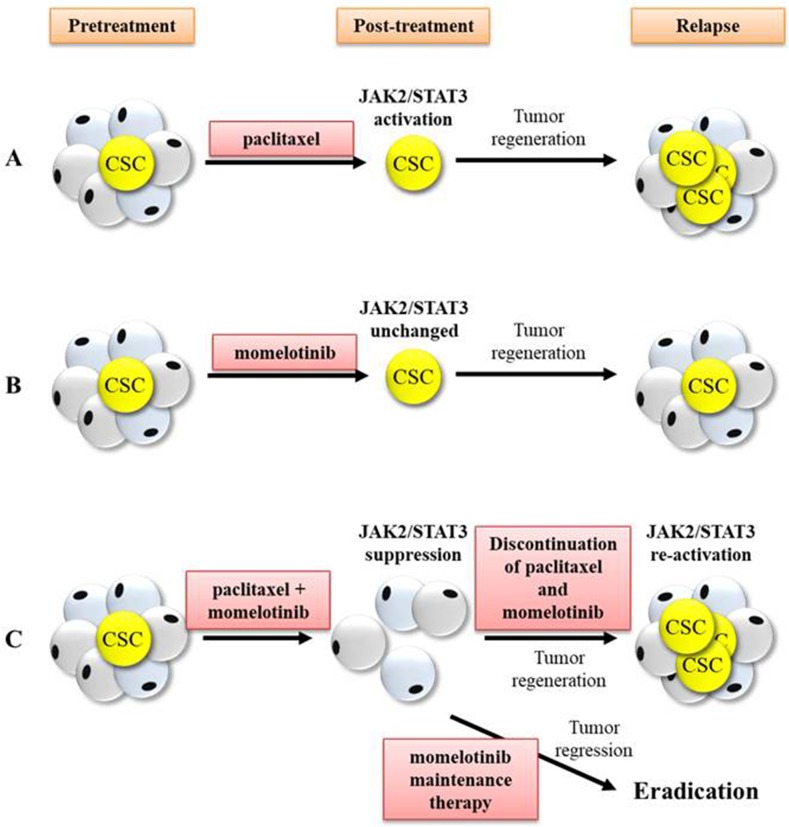
Proposed clinical outcome that may occur in response to treatment with paclitaxel, momelotinib, combination of paclitaxel and momelotinib, and momelotinib maintenance therapy CSCs are a small subset of tumor cells that are accountable for tumor regeneration and recurrence post-treatment. The JAK2/STAT3 signalling pathway is believed to be associated with the regulation of CSC-like development, thus targeting this pathway may eliminate CSCs and improve treatment outcome. **(A)** Treatment with weekly paclitaxel led to substantial reduction of tumor burden but increased JAK2/STAT3 activation and CSCs in the residual cells, thus resulting in tumor regeneration towards recurrence. **(B)** Treatment with daily momelotinib alone showed no effect on JAK2/STAT3 activation but failed to eliminate CSCs, resulting in tumor regeneration towards recurrence. **(C)** Treatment with a combination of paclitaxel and momelotinib effectively suppressed JAK2/STAT3 activation and targeted CSCs. However, the discontinuation of paclitaxel and momelotinib-combined treatment resulted in the re-activation of JAK2/STAT3 and re-establishment of CSCs, leading to tumor regeneration towards recurrence. On the other hand, maintenance therapy with daily momelotinib treatment resulted in tumor regression until to a point of total tumor eradication in 50% of mice.

This study highlights that the addition of momelotinib to current chemotherapy regimens may be a promising therapeutic approach for the treatment of advanced-stage ovarian cancer patients. In this context, momelotinib has recently undergone a Phase I/II clinical trial for myelofibrosis, a fatal myeloproliferative neoplasm driven by the constitutive activation of JAK2/STAT3 pathway frequently caused by a JAK2V617F gain-of-function mutation which involves a valine-to-phenylalanine modification at position 617 [52]. Ongoing treatment with momelotinib has shown low level of toxicity in myelofibrosis patients, and has also shown significant recuperation in spleen response rate with substantial improvement of symptoms within the first month of therapy [53]. Hence, this preliminary ‘proof of concept’ study necessitates comprehensive evaluation of momelotinib in Phase 2 clinical trials in chemonaive and relapsed ovarian cancer patients. The proposed clinical outcome of paclitaxel and momelotinib-mediated therapy in ovarian cancer patients is illustrated in Figure 12.

## MATERIALS AND METHODS

### Cell lines

The human ovarian HEY cell line was derived from a human ovarian cancer xenograft originally grown from a peritoneal deposit of a patient with moderately differentiated papillary cystadenocarcinoma of the ovary [54]. The human ovarian clear cell adenocarcinoma cell line TOV21G was derived from grade 3, stage III, primary malignant adenocarcinoma from a patient who was not exposed to chemotherapy or radiation therapy [55]. The HEY cell line was grown in complete RPMI 1640 (Sigma-Aldrich, Sydney, Australia) supplemented with 10% (v/v) heat inactivated foetal bovine serum (FBS) (Thermo Fisher Scientific, MA, USA), 2mM L-glutamine (Murdoch Childrens Research Institute, Victoria, Australia) and 1% (v/v) penicillin and streptomycin. The TOV21G cell line was maintained in 1:1 mixture of MCDB131 (Life Technologies, CA, USA) and Medium 199 (Lonza, Basel, Switzerland) supplemented with 15% (v/v) heat inactivated FBS (Thermo Fisher Scientific), 2mM L-glutamine and 1% (v/v) penicillin and streptomycin. Cells were routinely checked for mycoplasma infection.

### Antibodies and reagents

Polyclonal antibody against phosphorylated (Tyr-705) STAT3 (P-STAT3), total STAT3 (T-STAT3), phosphorylated (Tyr-1007/1008) JAK2 (P-JAK2), total JAK2 (T-JAK2), EpCAM and CD117 (c-Kit) were obtained from Cell Signalling Technology (Beverly, MA, USA). Monoclonal antibody against CD133 was obtained from Miltenyl Biotec (Macquarie Park, NSW, Australia). Antibodies against Ki67, CA125, Oct3/4 CD117 (c-Kit) and CD31 used for immunohistochemistry were obtained from Ventana (Roche, Arizona, USA). Momelotinib was obtained from Dr Christopher Burns, Walter and Eliza Hall Institute of Medical Research, Melbourne, Australia.

### Treatment of HEY and TOV21G cell lines with paclitaxel, momelotinib or a combination of both

Ovarian cancer HEY and TOV21G cell lines were treated with paclitaxel at a concentration at which 50% growth inhibition (GI_50_) was obtained (0.05μg/mL for HEY cells and 0.01μg/mL for TOV21G cells). The response of HEY and TOV21G cell lines in response to paclitaxel was determined by MTT assay at 24 hour as described previously [35]. This was to ensure that the surviving cells had been exposed to an adequate concentration of the drug, while ensuring sufficient cell numbers for *in vitro* analyses. For momelotinib treatment, cells were screened for the response to different concentrations of momelotinib in both cell lines, and the concentration that gave optimum inhibition of P-JAK2 and P-STAT3 in response to paclitaxel was selected in both cell lines (1μM for both cell lines).

### Immunofluorescence analysis

Immunofluorescence was performed as described previously [2, 20]. Fluorescence imaging was visualized and captured using an Olympus Cell^R^ fluorescence microscope. Semi-quantitative analysis to assess fluorescence intensity was performed using the inbuilt Cell^R^ software.

### SDS-PAGE and Western blot analysis

SDS-PAGE and Western blot was performed on the cell lysates as described previously [20].

### Flow cytometry analyses

Flow cytometry was used to assess the expression of cell surface markers as described previously [2, 3]. Data was analysed using Cell Quest software (Becton-Dickinson, Bedford, MA, USA).

### Quantitative real time PCR (qRT-PCR) analysis

RNA extractions, cDNA synthesis and quantitative determination of mRNA levels of various genes were performed as described previously [56]. The sequence of forward and reverse primers is described in Table 1. Real time PCR was performed using the Applied Biosystems ABI 7900 HT Fast real-time machine. Yields were converted to femtograms (fg) based on the standard curve for each product and the resulting mRNA levels were normalized to the 18S mRNA level per sample. Each experiment was performed in triplicate on three independent biological samples.

**Table 1:** Primer sequences of human oligonucleotides used in Quantitative Real-time PCR

Oligonucleotide Name	Forward (F) 5′ - 3′ Reverse (R) 5′ - 3′	Primer Sequence (5′ - 3′)	Size (bp)
EpCAM	F	CGT CAA TGC CAG TGT ACT TCA GTT G	301
	R	TCC AGT AGG TTC TCA CTC GCT CAG	
Oct4A	F	CTC CTG GAG GGC CAG GAA TC	381
	R	CCA CAT CGG CCT GTG TAT AT	
CD133	F	ATT GGC ATC TTC TAT GGT TT	167
	R	GCC TTG TCC TTG GTA GTG T	
18S	F	GTA ACC CGT TGA ACC CCA TT	153
	R	CCA TCC AAT CGG TAG TAG CG	

### Animal ethics statement

This study was carried out in strict accordance with the recommendations in the Guide for the Care and Use of the Laboratory Animals of the National Health and Medical Research Council of Australia. The experimental protocol was approved by the University of Melbourne's Animal Ethics Committee (Project-1413207.1), and was endorsed by the Research and Ethics Committee of Royal Women's Hospital Melbourne, Australia.

### Animal studies

Female Balb/c nu/nu mice (age, 6–8 weeks) were obtained from the Animal Resources Centre, Western Australia. Animals were housed in a standard pathogen-free environment with access to food and water. Animal experiments were performed on Balb/c nude mice as described below:

### Experimental design

Briefly, female Balb/c nu/nu mice (age 6 - 8 weeks) were injected ip with 5 × 10^6^ viable ovarian cancer HEY cells, a dose previously shown to produce significant tumor burden [3]. After 19 days, mice were treated weekly with ip injection of paclitaxel at a concentration of 15mg/kg of mice bodyweight with or without momelotinib given daily at a concentration of 25mg/kg of mice bodyweight by oral gavage. Mice were inspected every 2 - 3 days and tumor formation was monitored by palpation in addition to the overall body condition and body weight until the pre-determined endpoint was reached. Phase 1 and 2 studies were performed simultaneously, with a common control untreated group (n=4). At the endpoint, organs (such as liver, kidney, gastrointestinal tract, pancreas and spleen) and solid tumors were collected and processed for H&E and/or immunohistochemical analysis.

### Phase 1 study

Nineteen days post-ip injection of HEY cells, mice were divided into three groups based on the treatment given: paclitaxel, momelotinib, and a combination of paclitaxel and momelotinib (n=4 control and n=3/treatment group). All treatments were maintained until the endpoint of control untreated mice was reached. At this point, all mice in Phase 1 study were euthanized. Mice were monitored and weighed daily and any sign of tumor development was assessed by palpating the abdomen. Endpoint criteria included loss of body weight exceeding 20% of initial body weight and other physical parameters such as extension of abdomen anorexia and general patterns of diminished well-being such as abnormalities in respiration, motility, posture, and response to provocation [3, 20].

### Phase 2 study

Nineteen days post-ip injection with HEY cells, mice were divided into three groups based on the treatment given: paclitaxel, momelotinib, and a combination of paclitaxel and momelotinib (n=4-5/group of paclitaxel or momelotinib treated mice or combined paclitaxel and momelotinib treated mice). All treatments were maintained until the endpoint of control untreated mice. At this point, the control untreated mice were euthanized and all treatments in other groups were terminated. Mice in the treatment group were inspected every 2 – 3 days until their individual endpoint were reached as described in Phase 1 study.

### Phase 3 study

Nineteen days post-ip injection of HEY cells, all mice were treated with a combination of paclitaxel and momelotinib, except for the control untreated mice (n=9) and were divided into three groups (n=9/group). In Group 1, all treatments were maintained until the endpoint of control untreated mice. At this point, the control untreated and Group 1 mice were euthanized. In Group 2, all treatments were maintained until the endpoint of control untreated mice. At this point, all treatments were terminated but the treated mice were maintained and monitored until the end point was reached. In Group 3, all treatments were maintained until the endpoint of control untreated mice. At this point, only paclitaxel treatment was terminated while momelotinib only treatment was maintained by oral gavage. Mice in Group 3 were inspected every 2 – 3 days until their individual endpoint was reached, or until the designated endpoint of the experiment at 91 days post-inoculation.

### Immunohistochemistry of mouse tumors

Hemotaxylin & Eosin (H & E) staining of mouse organs and tumors was performed by the staff at the Anatomical Pathology Laboratory Services at The Royal Children's Hospital, Melbourne, Australia according to the standard H&E protocol [57]. Immunohistochemistry analysis of mouse tumors was performed on formalin fixed, paraffin embedded 4 μm sections of the xenografts and were stained using a Ventana Benchmark Immunostainer (Ventana Medical Systems, Inc, Arizona, USA) as described previously [3, 20]. Negative controls were prepared by incubating each tumor section without primary antibodies. Sections of human breast tissue, high-grade ovarian tumors and human tonsils were used as positive controls to determine the staining efficacy of primary antibodies. Immunohistochemistry images were taken using an Aperio ImageScope (Leica Microsystems, Mt Waverly, Australia) with the associated digital pathology viewing software. DAB staining was measured using the open source image processing package Fiji (https://fiji.sc/) with a plug-in developed to recognize DAB staining for 10 randomly captured images per section. DAB staining intensity of the negative control was subtracted from the DAB staining of the antibody of interest to get a measure of DAB staining of interest.

### Statistical analysis

All results are presented as the mean ± standard error of the mean (SEM) of three independent experiments unless otherwise indicated. Comparisons between more than two groups with one independent variable were analysed using One-Way ANOVA with Tukey's post hoc test. Comparisons of survival curves were analysed using Kaplan-Meier curves with Log-rank test. Each *in vitro* experiment was performed at least three times with a minimum of three biological replicates. Data was analysed using Microsoft Excel 2010 and GraphPad Prism Software (version 6). A probability level of p<0.05 was adopted throughout to determine statistical significance.

## SUPPLEMENTARY MATERIALS FIGURES



## Abbreviations

JAK2: Jak kinase 2
STAT3: signal transducer and activator of transcription 3
CSC: Cancer stem cells
IL-6: interleukin-6
GCSF: granulocyte colony stimulating factor
LIF: leukemia inhibitory factor
EGF: epidermal growth factor
CA125: cancer antigen 125
CD31: cluster of differentiation 31
EpCAM: epithelial cell adhesion molecule
CD133: promonin-1, encoded by PROM-1 gene
