# Supplementary material for: Momelotinib decreased cancer stem cell associated tumor burden and prolonged disease-free remission period in a mouse model of human ovarian cancer
Source: Oncotarget. 2018 Mar 30;9(24):16599–618. doi: 10.18632/oncotarget.24615 (PMC5908273; doi:10.18632/oncotarget.24615)
Supplement: Supplementary file 1 [file oncotarget-09-16599-s001.pdf]

Momelotinib decreased cancer stem cell associated tumor burden and prolonged disease-free remission period in a mouse model of human ovarian cancer

SUPPLEMENTARY MATERIALS

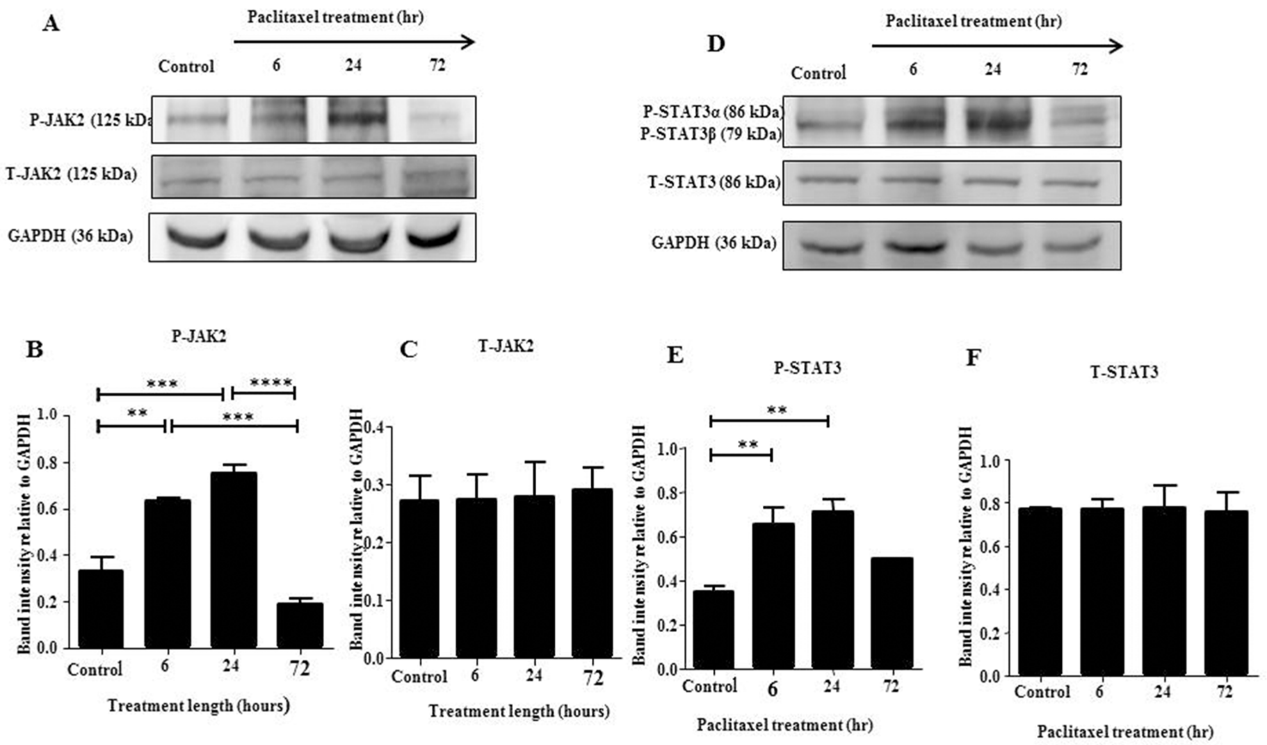

Supplementary Figure 1: JAK2 and STAT3 activation in TOV21G cells in response to paclitaxel treatment by Western blot. (A and D) Total cell lysates of untreated cells and cells treated with 0.01µg/mL of paclitaxel following 6, 24 and 72 hours of paclitaxel treatment were prepared and subjected to Western blot analysis using antibodies specific for P- or T-JAK2 and P- or T-STAT3. Total protein load was determined by stripping and re-probing the membranes with GAPDH. Images are representative of four independent lysate samples. Densitometric analysis of **(B-C)** P-JAK2 and T-JAK2, and **(E-F)** P-STAT3 and T-STAT3 protein expression was determined by using Image J. The values represent the relative mean band intensity normalized to GAPDH loading control ± SEM of four independent experiments. Parametric one-way ANOVA with Tukey's post-test was used. Significance is indicated by $^{**}p<0.01$, $^{***}p<0.001$, $^{****}p<0.0001$.

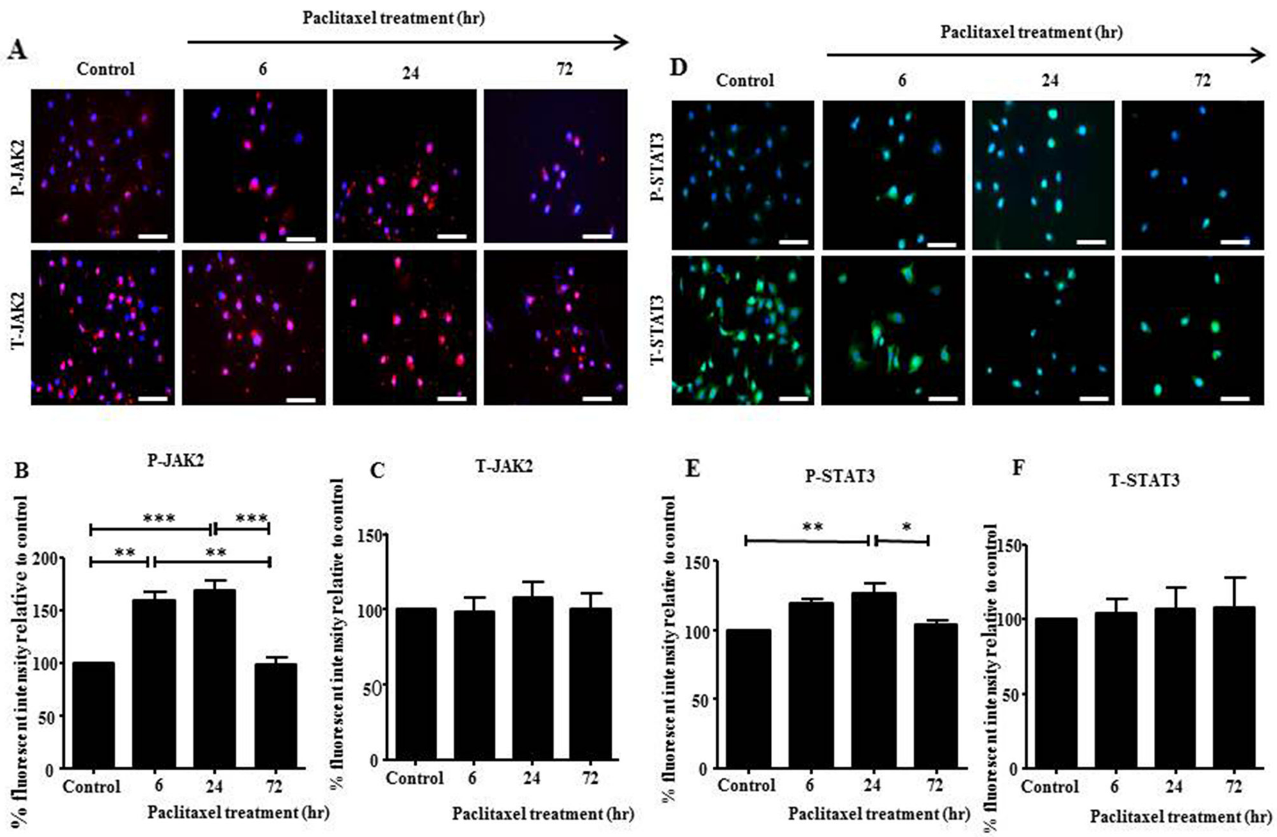

Supplementary Figure 2: JAK2 and STAT3 activation in TOV21G cells in response to paclitaxel treatment by immunofluorescence. (A and D) Expression and localization of activated P-JAK2 / STAT3 and T-JAK2 /STAT3 in ovarian cancer TOV21G cell line following 6, 24 and 72 hours treatment with paclitaxel were determined by immunofluorescence staining. Untreated and treated cells were assessed for the expression of P-JAK2, T-JAK2, P-STAT3 and T-STAT3 by immunofluorescence using rabbit and mouse polyclonal and monoclonal antibodies as described in the Materials and Methods section. Staining was visualized using the secondary Alexa 590 (red) and Alexa 488 (green) fluorescent-labelled antibodies and nuclei were detected by DAPI (blue) staining. Images are representative of three independent experiments. Magnification was 400X; scale bar = 250μm. Quantification of **(B-C)** P-JAK2 and T-JAK2, and **(E-F)** P-STAT3 and T-STAT3 fluorescent intensity was determined by using Image J. Results are expressed as the percentage of the average fluorescent intensity value relative to untreated cells ± SEM of three independent experiments. Parametric One-way ANOVA with Tukey's post-test was used. Significance is indicated by $^{*}p<0.05$, $^{**}p<0.01$, $^{***}p<0.001$.

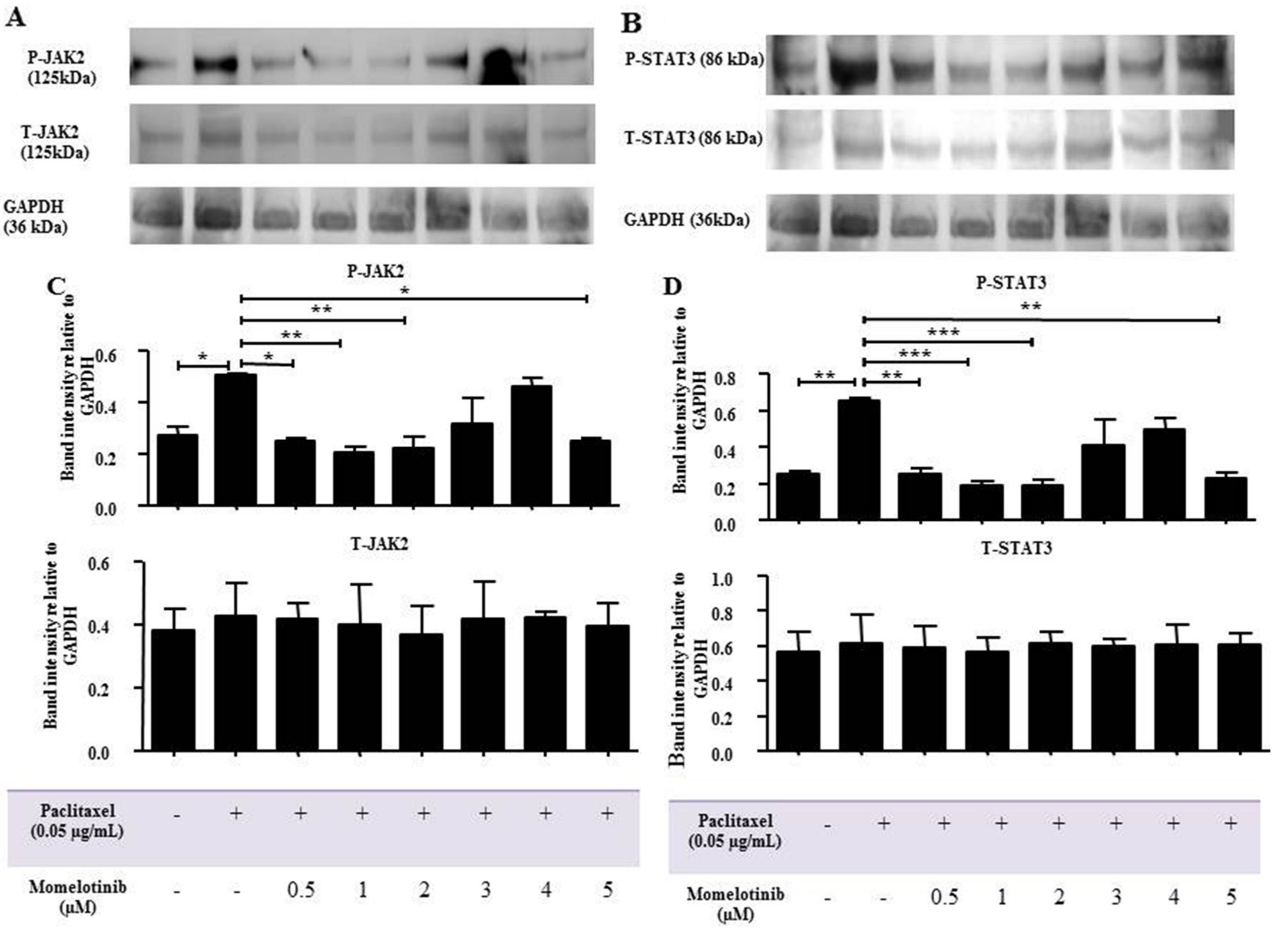

Supplementary Figure 3: Dose-dependent effect of momelotinib on paclitaxel-induced JAK2 and STAT3 activation in HEY cells. Optimal suppression of JAK2 and STAT3 activation in HEY cells treated with paclitaxel and increasing concentration of momelotinib was evaluated by Western blot. **(A-B)** Total cell lysates of untreated and cells treated with 0.05µg/mL paclitaxel and/or momelotinib at concentrations of 0.5 - 5µM for 24 hours were subjected to immunoblot analysis using antibody specific for P- or T-JAK2 and STAT3. Total protein load was determined by stripping and re-probing the membranes with GAPDH. Images are representative of three independent lysate samples. **(C-D)** Densitometric analyses of P-JAK2, T-JAK2, P-STAT3 and T-STAT3 protein expression were determined by using Image J. The values represent the relative mean of band intensity normalized to GAPDH loading control ± SEM of three independent experiments. Parametric One-way ANOVA with Tukey's post-test was used. Significance is indicated by $^{*}p<0.05$, $^{**}p<0.01$, $^{***}p<0.001$.

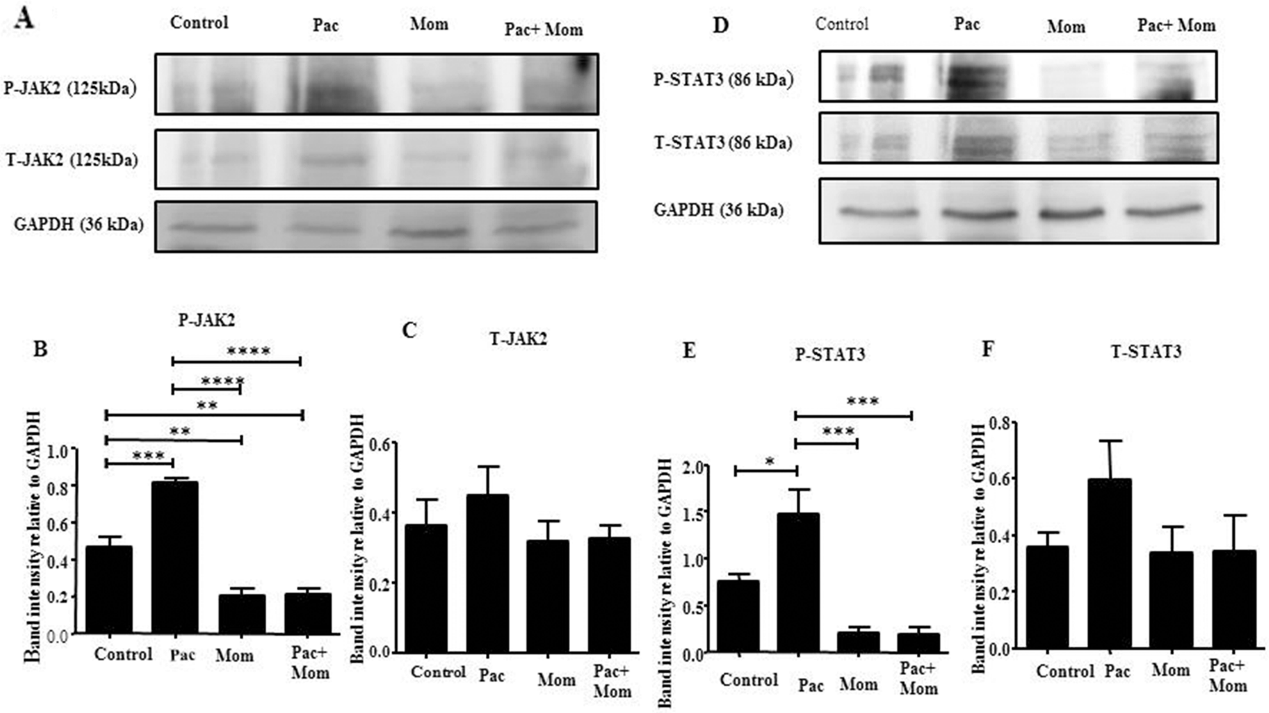

Supplementary Figure 4: Effect of paclitaxel and/or momelotinib treatment on the activation of JAK2 and STAT3 in TOV21G cells by Western blot. (A and D) Total cell lysates of untreated cells and cells treated with 0.01µg/mL of paclitaxel (Pac) with or without 1µM of momelotinib (Mom) for 24 hr were extracted and subjected to immunoblot analysis using antibody specific for P-JAK2/STAT3 or T-JAK2/STAT3. Total protein load was determined by stripping and re-probing the membranes with GAPDH. Images are representative of three independent lysate samples. Densitometric analyses of **(B-C)** P-JAK2 and T-JAK2, and **(E-F)** P-STAT3 and T-STAT3 protein expression were determined by using Image J. The values represent the relative mean of band intensity normalized to GAPDH loading control ± SEM of three independent experiments. Parametric One-way ANOVA with Tukey's post-test was used. Significance is indicated by $^{*}p<0.05$, $^{**}p<0.01$, $^{***}p<0.001$, $^{****}p<0.0001$.

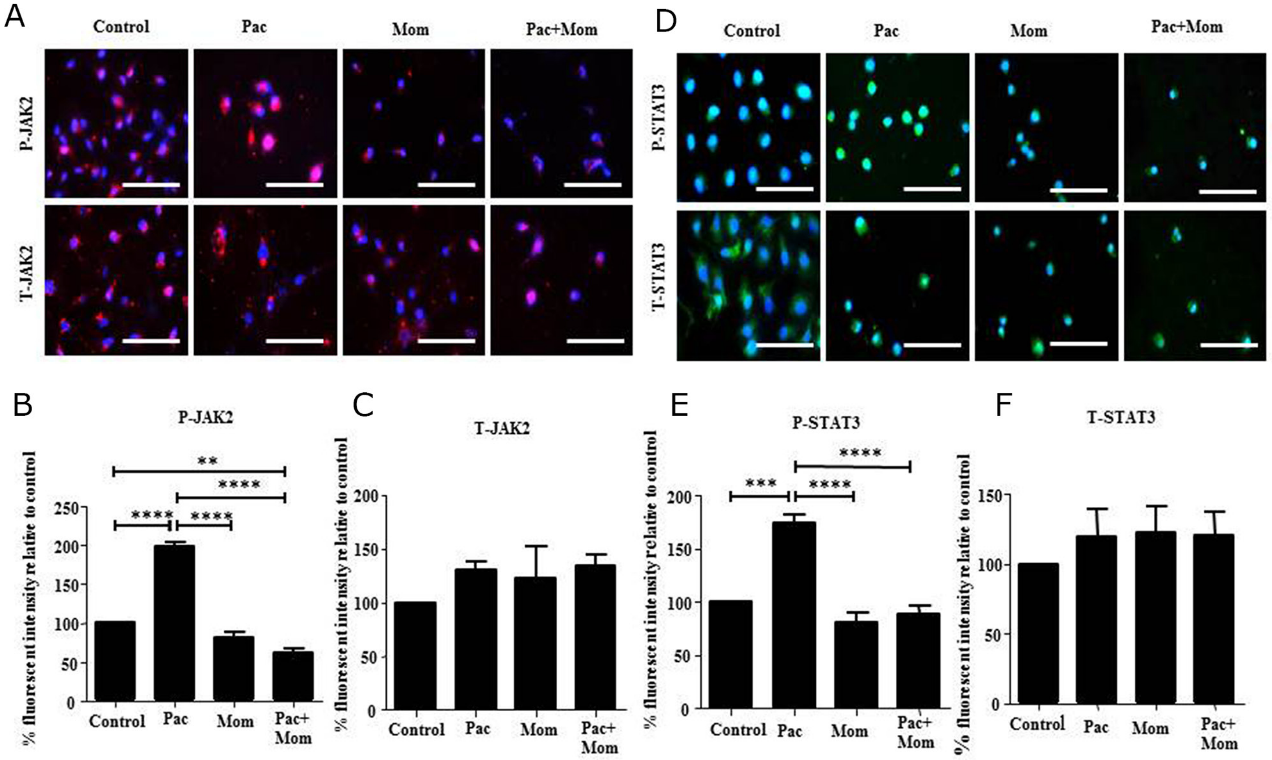

Supplementary Figure 5: Effect of paclitaxel and/or momelotinib treatment on JAK2 and STAT3 activation in TOV21G cells by immunofluorescence. (A-D) Expression and localization of the JAK2 and STAT3 activation in ovarian cancer TOV21G cells in response to treatment with 0.01µg/mL of paclitaxel (Pac) with or without 1µM of momelotinib (Mom) for 24 hours was determined by immunofluorescence staining. Untreated and treated cells were assessed for the expression of P-JAK2/STAT3 and T-JAK2/STAT3 using rabbit and mouse polyclonal and monoclonal antibodies as described in the Materials and Methods section. Staining was visualized using the secondary Alexa 590 (red) and Alexa 488 (green) fluorescent-labelled antibodies and nuclei were detected by DAPI (blue) staining. Images are representative of three independent experiments. Magnification was 400X; scale bar = 250µm. Quantification of **(B-C)** P-JAK2 and T-JAK2, and **(E-F)** P-STAT3 and T-STAT3 fluorescent intensity was determined using Image J. Results are expressed as the percentage of the average fluorescent intensity value relative to untreated cells ± SEM of three independent experiments. Parametric One-way ANOVA with Tukey's post-test was used. Significance is indicated by $^{**}p<0.01$, $^{***}p<0.001$, $^{****}p<0.0001$.

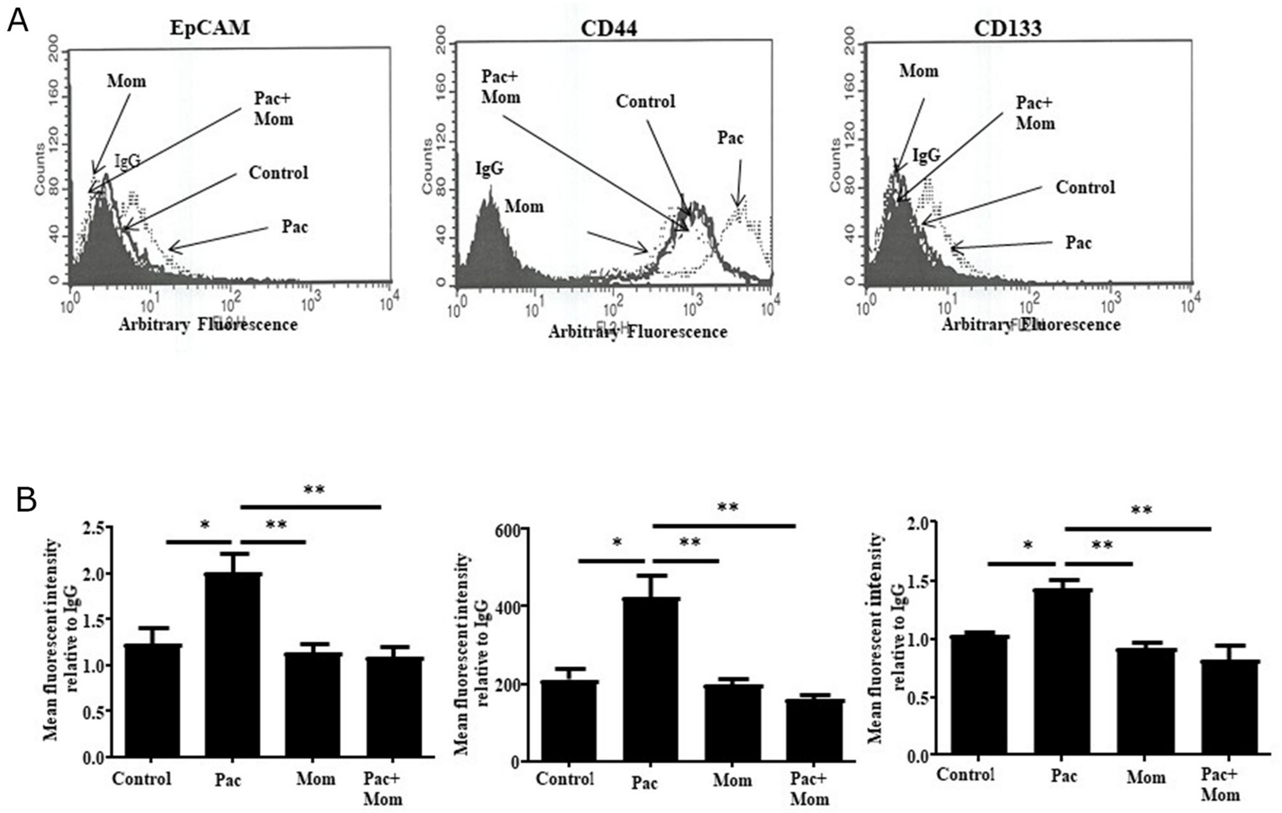

Supplementary Figure 6: Analysis of the expression of CSC-like surface markers in TOV21G cells treated with paclitaxel and/or momelotinib. (A) Cell surface expression of EpCAM, CD44 and CD133 in ovarian cancer TOV21G cells in response to a 24 hour treatments with 0.01µg/ml paclitaxel (Pac), 1µM momelotinib (Mom) or a combination of both was determined by flow cytometry. Untreated and treated cells were incubated with primary antibodies specific for EpCAM, CD44-PE, CD133-PE or IgG, followed by anti-mouse-PE secondary antibody for EpCAM detection. Histograms are representative of four independent experiments. **(B)** Semi-quantitative analysis of the arbitrary fluorescent expression of CSC-like markers was performed. Results are expressed as the mean arbitrary fluorescent expression of the markers of interest relative to negative control IgG ± SEM of four independent experiments. Parametric One-way ANOVA with Tukey's post-test was used. Significance is indicated by $^{*}p<0.05$, $^{**}p<0.01$.

Phase 2 Study

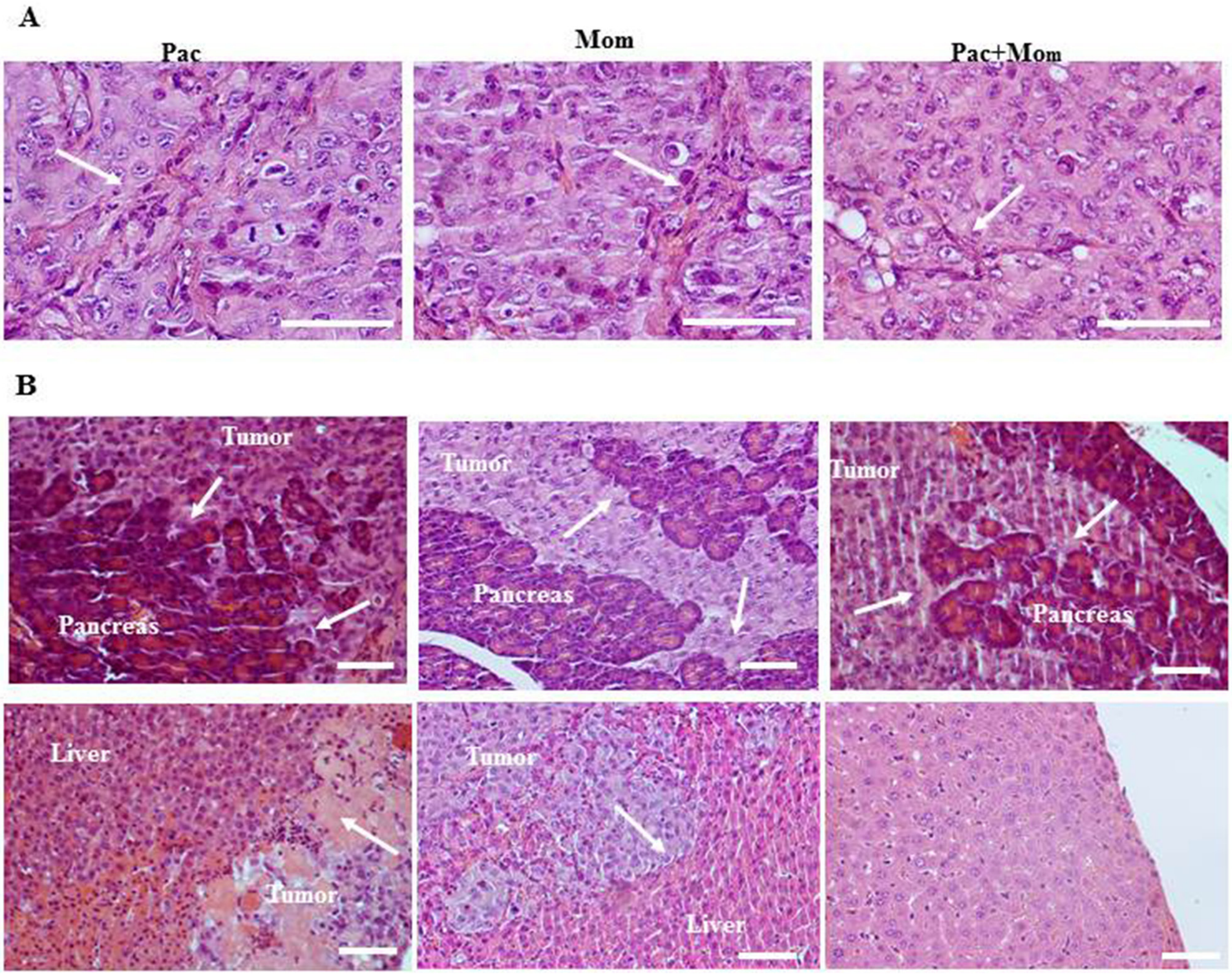

Supplementary Figure 7: Tumor histology of mice xenografts and infiltration in mice organs in Phase 2. (A) Representative images of H&E stained tumor xenografts derived from mice in Phase 2 study. Arrows indicate the presence of papillary projections. Magnification 400x, scale bar = 10μm. **(B)** Representative images of H&E stained pancreas and liver from Phase 2 (n=3/group). Arrows indicate tumor cells invading respective organs. Magnification 200x, scale bar = 10μm.

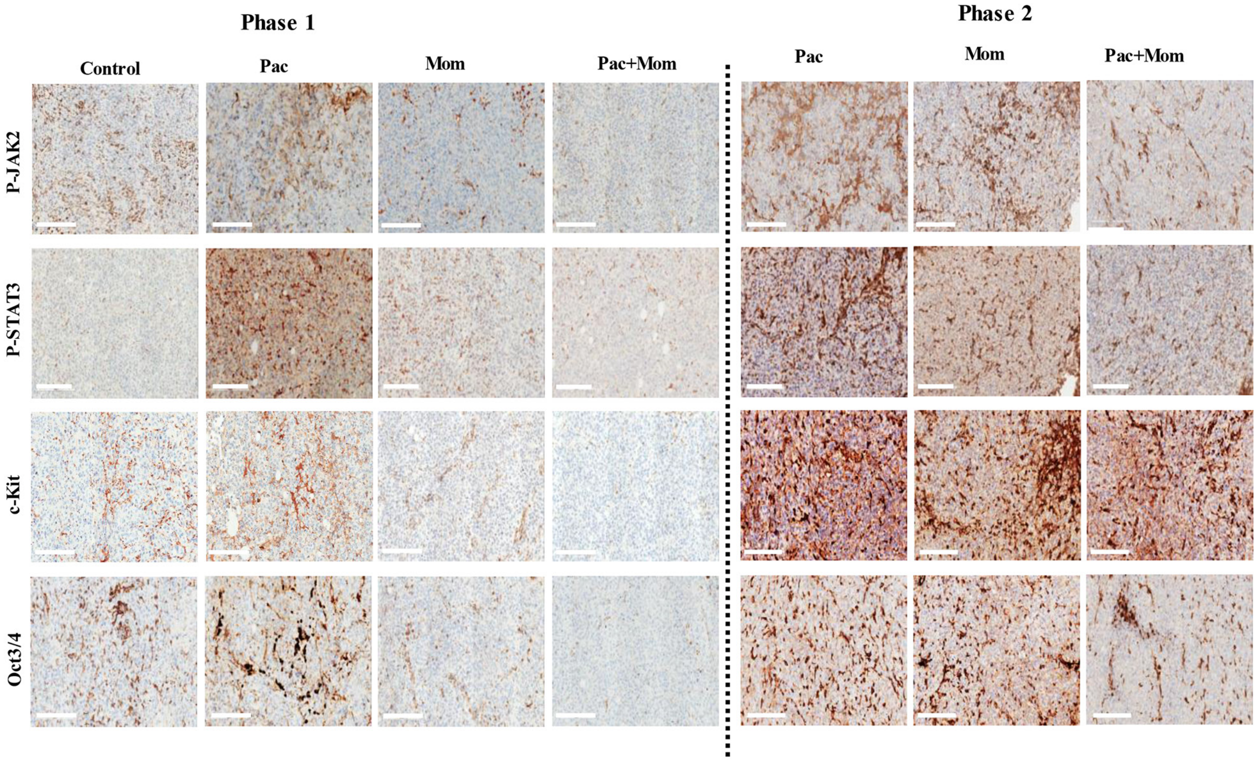

Supplementary Figure 8: Immunohistochemical comparison of P-JAK2, P-STAT3, c-Kit and Oct3/4 expression in tumor xenografts derived from mice intraperitoneally injected with HEY cells in Phase 1 and 2. Representative images of P-JAK2, P-STAT3, c-Kit and Oct3/4 immunohistochemistry staining in paraffin embedded tumor xenografts derived from control untreated, paclitaxel (pac), momelonib (Mom) and momelotinib (Pac+Mom) treated mice in Phase 1 and 2 studies. Magnification 200x, scale bar = 200µm.

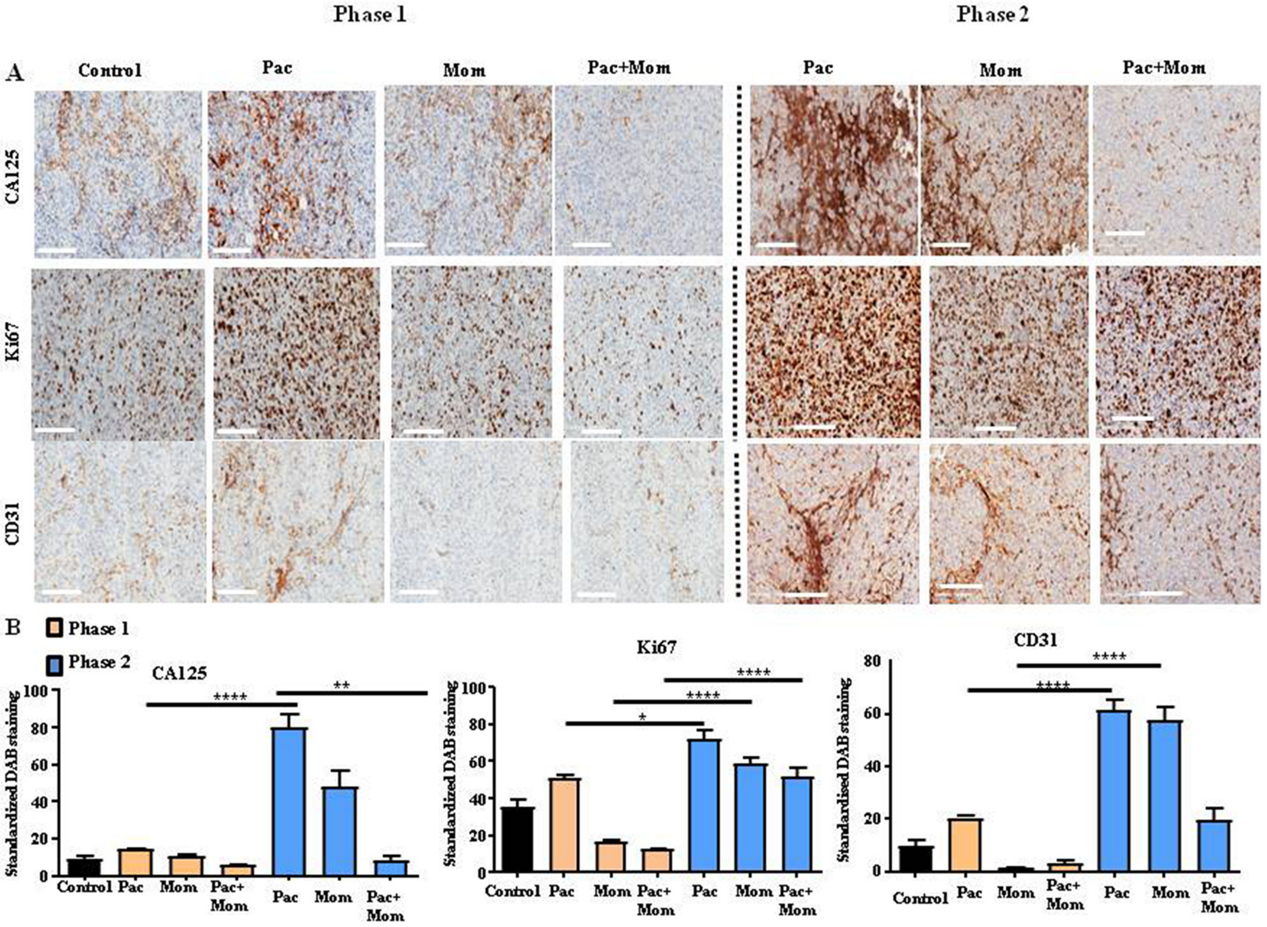

Supplementary Figure 9: Immunohistochemical comparison of CA125, Ki67 and CD31 in tumor xenografts derived from mice in Phase 1 and 2. **(A)** Representative images of CA125, Ki67 and CD31 immunohistochemistry staining in paraffin embedded tumor xenograft derived from mice in Phase 1 and 2. Magnification 200x, scale bar = 200µm. **(B)** Comparative quantification of CA125, Ki67 and CD31 DAB staining in mice tumor xenografts between Phase 1 and 2. Results are expressed as the average DAB reading of positively-stained tumor cells subtracted by the average DAB staining of the negatively-stained cells for each xenograft ± SEM (n=3/group). Parametric One-way ANOVA with Tukey's post-test was used to compare between the similar mice groups of Phase 1 and 2. Significance is indicated by *p<0.05, ****p<0.0001.

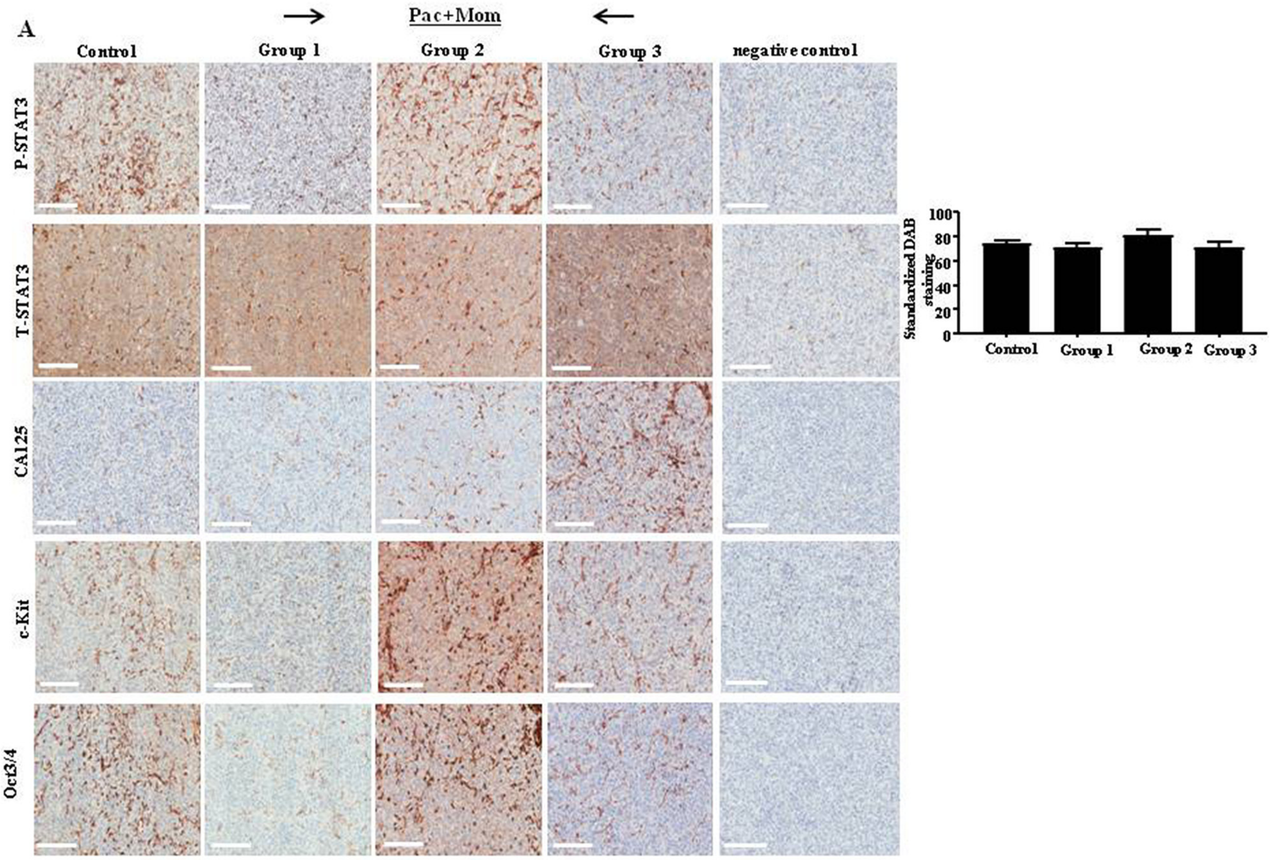

Supplementary Figure 10: Immunohistochemical comparison of P-STAT3, T-STAT3, CA125, c-Kit and Oct3/4 expression in tumor xenografts derived from mice intraperitoneally injected with HEY cells in Phase 3. **(A)** Representative images of P-STAT3, T-STAT3, CA125, c-Kit and Oct3/4 immunohistochemical staining in paraffin embedded tumor xenografts derived from control untreated, and Groups 1, 2 and 3 mice treated with paclitaxel and momelotinib (Pac+Mom). Magnification 200x, scale bar = 200µm. Quantification of T-STAT3, was done using DAB staining using Fiji software. Results are expressed as the average DAB reading of positively-stained tumor cells subtracted by the average DAB staining of the negatively-stained cells for each xenograft ± SEM (n=3/group). Parametric One-way ANOVA with Tukey's post-test was used for statistical analysis.
